# Comparative transcript profiling by SuperSAGE identifies novel candidate genes for controlling potato quantitative resistance to late blight not compromised by late maturity

**DOI:** 10.3389/fpls.2013.00423

**Published:** 2013-11-14

**Authors:** Astrid M. Draffehn, Li Li, Nicolas Krezdorn, Jia Ding, Jens Lübeck, Josef Strahwald, Meki S. Muktar, Birgit Walkemeier, Björn Rotter, Christiane Gebhardt

**Affiliations:** ^1^Department Plant Breeding and Genetics, Max Planck Institute for Plant Breeding ResearchCologne, Germany; ^2^GenXPro GmbHFrankfurt, Germany; ^3^Saka-Pflanzenzucht GmbH & Co. KGWindeby, Germany

**Keywords:** potato, late blight, transcript profiling, SAGE, marker-assisted selection

## Abstract

Resistance to pathogens is essential for survival of wild and cultivated plants. Pathogen susceptibility causes major losses of crop yield and quality. Durable field resistance combined with high yield and other superior agronomic characters are therefore, important objectives in every crop breeding program. Precision and efficacy of resistance breeding can be enhanced by molecular diagnostic tools, which result from knowledge of the molecular basis of resistance and susceptibility. Breeding uses resistance conferred by single *R* genes and polygenic quantitative resistance. The latter is partial but considered more durable. Molecular mechanisms of plant pathogen interactions are elucidated mainly in experimental systems involving single *R* genes, whereas most genes important for quantitative resistance in crops like potato are unknown. Quantitative resistance of potato to *Phytophthora infestans* causing late blight is often compromised by late plant maturity, a negative agronomic character. Our objective was to identify candidate genes for quantitative resistance to late blight not compromised by late plant maturity. We used diagnostic DNA-markers to select plants with different field levels of maturity corrected resistance (MCR) to late blight and compared their leaf transcriptomes before and after infection with *P. infestans* using SuperSAGE (serial analysis of gene expression) technology and next generation sequencing. We identified 2034 transcripts up or down regulated upon infection, including a homolog of the kiwi fruit allergen kiwellin. 806 transcripts showed differential expression between groups of genotypes with contrasting MCR levels. The observed expression patterns suggest that MCR is in part controlled by differential transcript levels in uninfected plants. Functional annotation suggests that, besides biotic and abiotic stress responses, general cellular processes such as photosynthesis, protein biosynthesis, and degradation play a role in MCR.

## Introduction

Plant resistance to microbial attacks manifests itself at three phenotypic levels. First, the entire plant species is immune against infection by all isolates of a microbial species, since pathogen entry into plant cells is blocked at a very early stage (non-host resistance) (Nürnberger and Lipka, 2005). Second, avirulent strains of the pathogen are contained at the site of infection by local cell death (hypersensitive response, HR) (Heath, 2000). This leads to an incompatible interaction between the pathogen and its plant host. Virulent strains overcome this barrier resulting in a compatible interaction. The HR can range from very few necrotic cells to large necrotic lesions. It is genotype specific and triggered by single genes for resistance (*R* genes), which interact directly or indirectly with effector molecules of specific strains of the pathogen (Ellis et al., 2000; Bent and Mackey, 2007). Third, the host plant is amenable to pathogen infection (compatible interaction) but reduced infection and/or multiplication rates of the pathogen lead to slower progression of pathogen induced disease. This type of resistance is quantitative, because it is controlled by multiple genetic as well as environmental factors. Quantitative resistance can be defined as the natural variation of a compatible host-pathogen interaction. The phenotypic distinction between HR and quantitative resistance is not always clear cut (Poland et al., 2009). The molecular genetic basis and the mechanisms of plant resistance to pathogens are being studied mainly in experimental systems, in which HR-type resistance and susceptibility are alleles of a single locus and segregate as Mendelian factors. These studies have established detailed models for how pathogens are recognized by the host plant, how this recognition is transmitted to the cell nucleus, where local and systemic defense responses are initiated by the transcriptional activation or repression of a large number of genes (Hammond-Kosack and Jones, 1996; Eulgem, 2005; Bent and Mackey, 2007; Koornneef and Pieterse, 2008). In contrast, the understanding of quantitative resistance at the molecular level is very limited. Genetic evidence and plausibility suggest that genes functional in pathogen recognition, defense signaling and defense responses are, at least in part, involved in quantitative resistance (Poland et al., 2009; Kou and Wang, 2010).

The improvement of crop plant genetic resistance to pests and pathogens is a major objective of plant breeding. In this respect, both *R* gene mediated resistance and quantitative resistance are important. Resistance factors are incorporated in advanced cultivars by introgression breeding, mostly from landraces or closely related wild species. However, in a number of pathosystems of worldwide relevance, *R* gene mediated resistance often proved not durable under field conditions (Wastie, 1991; Skamnioti and Gurr, 2009). *R* genes introgressed into varieties were soon defeated by new, virulent races of the pathogen, due to the fact that single mutations in the pathogen can overcome *R* gene mediated resistance. Such mutations are favored by strong selection pressure, when highly resistant varieties carrying single *R* genes are widely cultivated. Quantitative resistance is considered more durable due to its partial and polygenic nature, which exerts less selection pressure on the pathogen and requires more than one mutation to overcome resistance. However, by the same reasons, quantitative resistance is more difficult to analyze and to handle in breeding programs (Parlevliet, 2002; Poland et al., 2009).

The interaction of the oomycete *Phytophthora infestans* with the potato (*Solanum tuberosum*) causes late blight, the world wide most important disease in potato cultivation (Kamoun and Smart, 2005; Forbes, 2012). *R* genes introgressed during the 20th century from the wild potato species *Solanum demissum* proved not durable in the field, a fact that shifted the interest of potato breeders to quantitative resistance (Ross, 1986; Wastie, 1991; Colon et al., 1995). The phenotypic selection of cultivars with quantitative resistance requires multi-year and location trials and is complicated by the fact that quantitative resistance is often accompanied by other, negative agricultural attributes such as late plant maturity (Visker et al., 2004; Forbes, 2012). Plant maturity is a quantitative developmental trait influenced by day length (Kloosterman et al., 2013). It refers to the time the potato plant requires to complete its annual life cycle, beginning with sprouting, shoot/leaf growth, flowering, tuber initiation, and ending with tuber maturation and foliage senescence. Knowledge of the loci and their natural allelic variants that control quantitative resistance not compromised by late maturity will facilitate the selection of improved cultivars by means of DNA-markers, which are diagnostic for superior resistance or inferior susceptibility alleles.

Genetic dissection of quantitative resistance to late blight via molecular linkage mapping has identified a minimum of 20 quantitative trait loci (QTL) on the 12 potato chromosomes (Gebhardt and Valkonen, 2001; Simko et al., 2007; Danan et al., 2011). Most QTL for resistance to late blight detected so far were linked with QTL for plant maturity (Collins et al., 1999; Oberhagemann et al., 1999; Bormann et al., 2004; Visker et al., 2005; Danan et al., 2011). Some resistance QTL co-localized with *R* genes for resistance to *P. infestans* and other pathogens or with genes of unknown function that share high sequence similarity with *R* genes (RGL, resistance gene like), with genes functional in defense signaling and with genes involved in defense responses (Leonards-Schippers et al., 1994; Leister et al., 1996; Gebhardt and Valkonen, 2001; Trognitz et al., 2002; Pajerowska et al., 2005). The genetic linkage studies collectively support the idea that natural variation in genes that control *R* gene mediated resistance is causal for quantitative resistance to *P. infestans* and other pathogens. They formed the basis of a candidate gene approach toward the identification of DNA polymorphisms associated with quantitative resistance to late blight in multi-parental populations of potato varieties and breeding clones (Gebhardt et al., 2004; Malosetti et al., 2007; Pajerowska-Mukhtar et al., 2009). In particular, association mapping discovered single nucleotide polymorphisms (SNPs) in two potato genes coding for allene oxide synthase (*StAOS2* and *StAOS1*), which were associated with resistance to late blight not compromised by late maturity (Pajerowska-Mukhtar et al., 2009). Allene oxide synthase is a key enzyme in the biosynthesis of jasmonates, a class of lipid-derived plant hormones involved in defense and stress responses as well as developmental processes (Kombrink, 2012). Two neighboring SNP markers, StAOS2-snp691 and StAOS2-snp692 at the *StAOS2* locus on potato chromosome XI explained between thirty and forty percent of the genetic variation of maturity-corrected resistance to late blight (MCR) in a population of 184 breeding clones (Pajerowska-Mukhtar et al., 2009). Silencing the expression of *StAOS2* resulted in increased susceptibility to *P. infestans*. This result together with quantitative functional complementation analysis of five natural *StAOS2* alleles in an *aos* knock-out mutant of *Arabidopsis thaliana* provided evidence that DNA variation at the *StAOS2* locus is responsible, via a yet unknown mechanism, for a portion of the quantitative resistance to *P. infestans* (Pajerowska-Mukhtar et al., 2008). The role of *AOS* in quantitative resistance was not restricted to the pathosystem *S. tuberosum/P. infestans*, as overexpression of a rice *AOS* increased resistance of transgenic plants to the rice blast fungus *Magnaporthe grisea* (Mei et al., 2006).

Although successful, the candidate gene approach is necessarily biased toward genes with known function in pathogenesis and defense. To discover additional, novel genes involved in quantitative resistance, unbiased approaches such as comparative transcript, protein, or metabolite profiling are required. Few comparative transcript profiling studies addressed aspects of quantitative resistance to late blight in potato using subtractive hybridization approaches (Ros et al., 2004; Henriquez and Daayf, 2010) or cDNA microarrays (Restrepo et al., 2005; Wang et al., 2005; Lindqvist-Kreuze et al., 2010). In these studies, known and novel transcripts were identified that were up or down regulated upon infection with *P. infestans* in a compatible interaction. In most cases two different genotypes with different levels of resistance to late blight were compared. Such a comparison does not allow to distinguish whether transcripts are differentially expressed due to different resistance levels or due to unspecific differences between individual genetic backgrounds that are unrelated with the resistance phenotype. SuperSAGE (serial analysis of gene expression) combined with next-generation sequencing is a novel platform for capturing and quantifying in depth and *de novo* the transcriptome in a biological sample (Matsumura et al., 2003, 2008; Gilardoni et al., 2010). SuperSAGE generates 26 base pair sequence tags from each transcript in the sample. The tag frequency is proportional to the amount of the corresponding transcript in the sample. Comparisons of tag frequencies between pairs of samples reveal differences in expression level. Mapping the tag sequences to collections of expressed sequences and to annotated genome sequences allows annotation and thereby the identification of putative functions of the corresponding transcripts.

Here we describe the results of a comprehensive transcript profiling experiment using SuperSAGE technology. The plants analyzed resulted from marker-assisted selection for increased vs. decreased quantitative resistance to *P. infestans* not compromised by late plant maturity. Our objectives were (i) the validation of the diagnostic power of SNP markers at the *StAOS2* locus that were previously shown to be associated with maturity-corrected resistance (MCR) to late blight and (ii) the identification of novel potato candidate genes for having a functional role in quantitative resistance to *P. infestans*.

## Materials and methods

### Plant material and marker assisted selection (MAS)

Based on prescreening tetraploid, heterozygous parental clones with the SNP markers StAOS2-snp691 and StAOS2-snp692, two half-sib families derived from the crosses Phy20 × Phy14 and Phy20 × Phy16 were chosen for MAS. The parents Phy14, Phy16, and Phy20 were breeding clones from the commercial breeding program of SaKa Pflanzenzucht GmbH & Co. KG (Windeby, Germany). The common seed parent Phy20 (genotype: StAOS2-snp691: *AAGG*; StAOS2-snp692: *CCGG*) was maturing mid-early (score 5) and was more susceptible to late blight than the pollen parents Phy14 and Phy16 (MCR = 64.2). The pollen parents Phy14 (genotype: StAOS2-snp691: *AAAA*; StAOS2-snp692: *CCCC*) and Phy16 (genotype: StAOS2-snp691: *AAAG*; StAOS2-snp/692: *CCCG*) were maturing mid-early (score 5) and mid-early to late (score 6), respectively, and expressed good maturity corrected resistance (MCR) to late blight (Phy14: MCR = −57.4; Phy16: MCR = −39.2). One tuber per F_1_ seedling was harvested in 2007 and planted in the field at Windeby (Germany) in 2008. Approximately 100 mg fresh leaf tissue (one young leaflet) per genotype were collected in 96 racked collection tubes (Qiagen, Hilden, Germany) and freeze dried. Genomic DNA was extracted from freeze dried leaf tissue using a BioSprint 96 robotic workstation (Qiagen, Hilden, Germany) according to the manufacturer's protocol. Plants were genotyped in 2008 for the SNP markers StAOS2-snp691 and StAOS2-snp692 by amplicon sequencing as described previously (Pajerowska-Mukhtar et al., 2009). Three genotype groups A1, A2, and B2 were selected. Marker-selected plants (SL genotypes) were tuber propagated in 2009 under standard phytosanitary regimes and re-genotyped using newly isolated DNA from field grown plants. Only genotypes with identical marker scores in both years were used for preparing SuperSAGE samples.

### Field evaluation of maturity corrected resistance (MCR) to late blight

Marker selected SL genotypes were evaluated in 2010 in separate field trials for plant maturity and resistance to late blight as described (Bormann et al., 2004; Pajerowska-Mukhtar et al., 2009). Briefly, plant maturity was scored from 1 to 9 (1 very early, 9 very late maturing) in comparison to standard varieties of known maturity type. Two replications, each with 12 plants per genotype (lattice design with partial blocks), were infected on 5th July 2010 with a mixture of complex *P. infestans* field isolates (provided by Julius Kühn Institutes Braunschweig and Groß-Lüsewitz, Germany). Using mixtures of *P. infestans* isolates insured that all major genes for late blight resistance potentially present in the plants were overcome. Disease progression was monitored by scoring the plants every 3 to 4 days from 12th July to 19th August, using a 0–9 scale (0: no symptoms, 9: plants are dead). Maturity corrected resistance (MCR) was calculated from the regression curve of the “area under disease progress curve” (AUDPC) against plant maturity. MCR corresponded to the vertical distance of each AUDPC value to the regression curve. Negative MCR values indicate higher levels of resistance, whereas positive MCR values indicate higher susceptibility (Bormann et al., 2004).

### Preparation of tissue samples for SuperSAGE and qRT-PCR

Twenty nine SL genotypes were multiplied by shoot cuttings. Six to seven weeks old plants (three plants per SL genotype) were transferred to a growth chamber (Snijders Scientific B.V., Tilburg, Netherlands) and acclimated for few days to 16 h light at 22°C and 8 h dark at 20°C before infection with *P. infestans*. The same mixture of *P. infestans* isolates as used for field inoculations (see above), which included all 11 race specificities were used for infection experiments. The race composition was confirmed by a detached leaflet test (Goth and Keane, 1997) using the race 1–11 differential set of potato cultivars from the James Hutton Institute (former Scottish Crop Research Institute, Invergowrie, UK). *P. infestans* was propagated on detached leaflets of 6–10 weeks old susceptible plants (cvs. Désireé or Grata) or on tuber slices of the susceptible variety Grata. Inoculum was prepared as described (Gyetvai et al., 2012). Three compound leaves per plant (3rd, 4th, and 5th from the top) were spray inoculated on the abaxial side each with 200–400 μl sporangia suspension (20,000 sporangia/ml, 65,000–130,000 zoospores/ml) using a pump glass flask. The plants were then covered with clear plastic bags to insure high humidity for optimal growth of *P. infestans*. Inoculations and tissue sampling were performed between 8:00 and 11:30 A.M. Individual leaflets of similar size were collected from the 4th and 5th compound leaf just before inoculation (T0), one (T1) and two (T2) days post inoculation. With these infection time points we captured two time slots from the mid to late biotrophic growth phase of *P. infestans* (Avrova et al., 2003). We assumed that transcriptome differences during the biotrophic phase will be more informative than during the necrotrophic phase. Leaflets were immediately frozen in liquid nitrogen. The 3rd compound leaf served as infection control. Infection symptoms were observed between the fourth and seventh day post inoculation. For each time point, leaf tissue was harvested from a different plant to avoid wounding and priming effects. The infection experiment was repeated five times, each with new batches of plants and inoculum. The best three infection experiments (experiment 1, 2, and 3) were chosen for qRT-PCR experiments (see below). Experiment 1 was used for constructing SuperSAGE libraries.

### SuperSAGE library construction and sequencing

SuperSAGE libraries were constructed from total RNA of nine leaf samples from infection experiment 1. For each time point (T0, T1 and T2) one leaflet each of similar size from 14, 6, and 9 SL genotypes in genotypic groups A1, A2, and B2 (Table 1), respectively, were pooled. Frozen pooled leaf tissue was powdered in a CryoMill (Retsch, Haan, Germany). Total RNA was extracted from powdered tissue using the Plant RNA Isolation Kit of Invitrogen (Karlsruhe, Germany) following the manufacturer's protocol. RNA purification, removal of genomic DNA contamination, concentration measurements and quality tests were performed as described (Draffehn et al., 2010). RNA integrity was additionally assessed by electrophoresis using an Agilent 2100 Bioanalyzer (Agilent Technologies, Santa Clara, USA). SuperSAGE libraries were prepared at GenXPro GmbH (Frankfurt, Germany) essentially as described (Matsumura et al., 2010). To prevent amplification biases the TrueQuant technology was applied as described by B. Rotter (Patent application Nr. WO2009152928). Two libraries were constructed for each RNA sample, one using *Nla*III (recognition site: 5′-CATG-3′) and the other using *Dpn*II (recognition site: 5′-GATC-3′). Transcripts that do not have a *Nla*III restriction site close to the 3′ end cannot be detected as no tag is generated. Those transcripts may be rescued by preparing libraries using a second anchoring enzyme. Therefore, the 26 bp tags carry either CATG or GATC at their 5′ end. The 18 libraries (3 genotype pools, 3 time points, 2 restriction enzymes) were pooled and sequenced by Solexa/Illumina technology on an Illumina GAII instrument (Illumina, Inc., USA). The sequence data analyzed in this paper have been deposited in NCBI's Gene Expression Omnibus (Edgar et al., 2002; Barrett et al., 2013) and are accessible through GEO Series accession number GSE48071 (http://www.ncbi.nlm.nih.gov/geo/query/acc.cgi?acc=GSE48071).

**Table 1:** **Summary of marker-assisted selection for MCR**.

**Cross combination**	**Phy20 × Phy14**	**Phy20 × Phy16**	
No. of F_1_ plants genotyped	309	142	
No. of F_1_ plants selected	14	6	9
Genotype *StAOS2-snp691*	*AAAA*	*AAAA*	*AGGG*
Genotype *StAOS2-snp692*	*CCCC*	*CCCC*	*CGGG*
Genotype group name	A1	A2	B2
No. of genotypes evaluated in the field	11^c^	4	8
Mean MCR	26.2^b^	−69.8^a^	5.9^b^

a,b Post-hoc test (LSD) for significant differences between groups.

c Some of the selected genotypes were eliminated before the field evaluation due to viral infections.

### Data analysis

The primary sequence data were purified as described (Matsumura et al., 2010). Tag counts were normalized (tags per million = tpm). The 26 bp tags were annotated by BLAST analysis (Altschul et al., 1990) using the databases STGI.042210 for potato transcripts (Ronning et al., 2003) and /NCBI/ENTREZ/NCBI_P.infestans_mRNA+REFSEQAUG10/NCBI_P.infestans_mRNA+REFSEQ.Fasta, BROAD_Ins/phytophthora_infestans_t30-4_1_genes.fasta, and BROAD_Ins/phytophthora_infestans_t30-4_1_transcripts.fasta for transcripts of *Phytophthora infestans*. For tag annotation, at least 20 bp of 26 bp total had to be identical with the sequence of the target transcript. Different tags matching to the same transcript were totaled to obtain absolute and normalized “hit” counts (hits per million = hpm). Likelihoods for different tag and hit counts in pair wise comparisons were calculated according to (Audic and Claverie, 1997). For mapping tags to the potato genome sequence (Xu et al., 2011), 266,361 unique tags (unitags) with more than five counts across all samples were extracted from the SuperSAGE output files and converted into FASTA format. Tag mapping was performed in forward and reverse orientation with the BWA (Burrows-Wheeler Alignment) software (Li and Durbin, 2009) (http://bio-bwa.sourceforge.net/). Tags were mapped against the annotated genome of *Solanum phureja* Group *tuberosum* (PGSC_DM_v3_2.1.11_pseudomolecule_annotation.gff) allowing either no mismatch or up to three base pairs mismatches. BWA output files were filtered for tags with alignment quality scores larger than 20. Venn diagrams were constructed using the package VennDiagram v.1.5.1 of the statistical software R v.2.15.1 (Venables et al., 2008). Differentially expressed tags (*p* < 10^−4^) of fifteen pair wise comparisons were visualized.

### *In silico* mapping of DNA-markers and transcripts

Restriction fragment length polymorphism (RFLP) markers linked to QTL for resistance to late blight and other pathogens were collected from the literature (Leonards-Schippers et al., 1994; Zimnoch-Guzowska et al., 2000; Ballvora et al., 2011; Danan et al., 2011). Potato marker sequences were obtained from the PoMaMo (Potato Maps and More) database at http://www.gabipd.org/ (Meyer et al., 2005) and tomato marker sequences from the SOL genomics network (SGN) at http://solgenomics.net/. Sequences of markers and potato transcripts were matched by BLAST (Altschul et al., 1990) to the potato pseudomolecules (version v2.1.11) (Xu et al., 2011) at http://solanaceae.plantbiology.msu.edu/integrated_searches.shtml, to retrieve genomic positions and the corresponding loci. At least 90% sequence identity had to be reached between query and target sequence.

### Quantitative real time qRT-PCR

cDNA synthesis and qRT-PCR were performed as described (Draffehn et al., 2010, 2012). Primer sequences, amplicon lengths, and annealing temperatures are listed in Table 2. Whenever possible, one primer was designed such that it included all or part of the differential SuperSAGE tag with the best match to the target transcript, to promote locus specific amplification. PCR conditions for all primer pairs were: Initial denaturation at 95°C for 10 min followed by 50 cycles of 95°C for 15 s, annealing at the temperatures listed in Table 2 for 30 s and extension at 72°C for 45 s. At the end of the PCR, reactions were tested for undesired primer dimer formation by melting curve analysis (55–95°C with a heating rate of 0.1°Cs^−1^ and continuous fluorescence measurement). In preliminary experiments the observed variation between technical replicates was small compared to the variation between different infection experiments. Average transcript levels were calculated from two technical replicates of each sample (three genotype pools at T0, T1, and T2) of three infection experiments. Expression levels were normalized against transcripts of the *SAND* gene (Czechowski et al., 2005; Exposito-Rodriguez et al., 2008) using the primer combination 5′-CTGCTTGGAGGAACAGACG-3′ and 5′-GCAAACAGGACCCCTGAATC-3′. PCR was carried out at 58°C annealing temperature and using otherwise the same conditions as above. Amplification of *SAND* resulted in a 164 bp fragment. Genotype pools were tested for successful infection using as internal marker the *P. infestans* transcript for the 60 s ribosomal protein L23a (PITG_02694) (Table 2). The *P. infestans* transcript values were not normalized against *SAND*.

**Table 2:** **Primers used for semiquantitative qRT-PCR**.

**Transcript**	**Annotation**	**Locus PGSC0003DMG**	**Chromosome**	**Forward (F) and reverse (R) primers (5′ to 3′)**	**Amplicon length (bp)**	**T_**a**_ (°C)**	**Differential tag included in primer**
TC194752	Putative membrane protein	400020757	V	F: GTTGGCGTTGCTGGATGGG	150	65	no
				R: GTGGTCTGGCTTTGGCCTCAG			
TC194940	Osmotin-like protein	400003057	VIII	F: AGAGGTGGGTGCCAGACCG	550	62	yes
				R:ACAAGAAAGAGGTGGCTACACTCATG			
TC195330	Ethylene response factor 5	400010753	XII	F: AGGTGCCGCCACTGTCAC	160	57	yes
				R:AATGAAATTACAAAATATTGAAGATC			
TC195401	CCR4 associated factor 1-related protein	400005899	VI	F: TCTGTAGACCGGGCGGT	140	55	yes
				R: CTAATCCATACAAGACGCCAG			
						
TC197025	Kiwellin	400008101	XI	F: CGATGACCCTGACGTTGG	130	60	no
				R: GACCACCCCCGTCTCCAC			
TC199390	1,3-beta-glucan glucanohydrolase	400020017	I	F: GCAATCGGTGAAGCTGGTTTGG	170	65	no
				R: ACGAGCAAAGGTGCACGCG			
TC200652	Caffeoyl-CoA O-methyltransferase	400006214	X	F:ATCGGTAGTGGCAGCACCTGATG	150	65	yes
				R: GCGGCGACACAAGGTGATGC			
TC204989	Lipoxygenase	400010859	VIII	F: CTCCCAAATCGCCCAACG	300	58	yes
				R: TCACCATTCATCTGTATAATTTGATCC			
TC206389	ZPT2-13	400015533	XI	F: CATGAGGCGTCATCGCG	155	60	yes
				R: CAATTGGTGCGGTCGGC			
TC208021	Jasmonate ZIM-domain protein	400002930	XII	F: GAGCTGTGGAAAGGTGTCAGAGC	160	58	yes
				R: CATAGTTAGATCAAAGTGGCT TCAATC			
TC209317	Non-specific lipid-transfer protein	400012481	VIII	F: GCCACCCCCAGTCACG	167	55	no
				R: GTGAAACAAACAACAATACATGTA			
TC212020	2-oxoglutarate-dependent dioxygenase	400011751	VII	F: CACGCATCATTGGAGCCC	176	59	yes
				R: TTAGGAAAGAAAGATTCTTAT TCATG			
						
TC218213	Lipid desaturase	400035878 or 400036004	?	F: CACCACACTCACTCTTCATTGC	190	58	no
				R: GCTTTTGTTGCCTCAACTGC			
TC221793	Xyloglucan-endotransglucosylase	400000408	XI	F: GGTCATGTTCACTTGCCATC	90	55	no
				R: CAAATTGCTCCGGGATTG			
TC225046	Conserved gene of unknown function	400027566	IV	F: AATACTACGGCGGCTGCG	150	57	yes
				R: CATATATATATAATTCCAAAGACATG			
PITG_02694	*P. infestans*, T40-3 60S ribosomal protein L23a	–	–	F: CGCCTGACCGCTGACTACGA	164	58	yes
				R: GCGAGAGTGCGATGACGATG			
						

The TC identifiers according to The Solanum tuberosum Gene Index (http://compbio.dfci.harvard.edu/cgi-bin/tgi/tc_ann.pl?gudb=potato), annotation, locus identifier according to PGSC (http://potatogenomics.plantbiology.msu.edu/index.html), chromosome, primers, amplicon length, annealing temperature (T_a_) and information about the integration of the significant SuperSAGE tag in one of the primer of the primer pair are given.

? indicates that no genomic position is available for this locus.

## Results

### Marker-assisted selection (MAS) for maturity-corrected resistance (MCR) to late blight

Four hundred and fifty one tetraploid, heterozygous F_1_ plants from two half-sib families were subjected to genotypic selection with the two neighboring SNP markers StAOS2-snp691 and StAOS2-snp692. The haplotype *StAOS2_A*_*691*_*C*_*692*_ was associated with increased resistance (negative MCR values) in a dosage dependent manner, whereas the haplotype *StAOS2_G*_691_*G*_692_ was associated with increased susceptibility (positive MCR values) (Pajerowska-Mukhtar et al., 2009). Fourteen and six genotypes were selected from 309 F_1_ plants of the family Phy20 × Phy14 and 142 F_1_ plants of the family Phy20 × Phy16, respectively, which were homozygous for haplotype *StAOS2_A*_*691*_*C*_*692*_ (Table 1). Plants homozygous for the haplotype *StAOS2_G*_691_*G*_69_, were not present in the F_1_ families due to the parental genotypes. As contrasting genotype group we selected nine genotypes of the family Phy20 × Phy16, which carried three copies of haplotype *StAOS2_G*_691_*G*_692_ and one copy of haplotype *StAOS2_A*_*691*_*C*_*692*_ (Table 1). Subsequently we refer to the three selected genotype groups as A1, A2, and B2. Groups A1 and A2 were identical with respect to the *StAOS2_A*_*691*_*C*_*692*_ haplotype but differed in genetic background as the plants originated from different cross combinations, whereas groups A2 and B2 originated from the same cross but differed with respect to the genotype at the *StAOS2* locus. The field evaluation of MCR of plants in groups A1, A2, and B2 showed that plants in group A2 were on average more resistant than plants in group B2. However, plants in group A1 were on average as susceptible as plants in group B2, irrespective of the fact that the A1 plants possessed the *StAOS2* SNP haplotype associated with higher resistance (Table 1).

### Comparative transcript profiling by SuperSAGE

**Figure 1:**
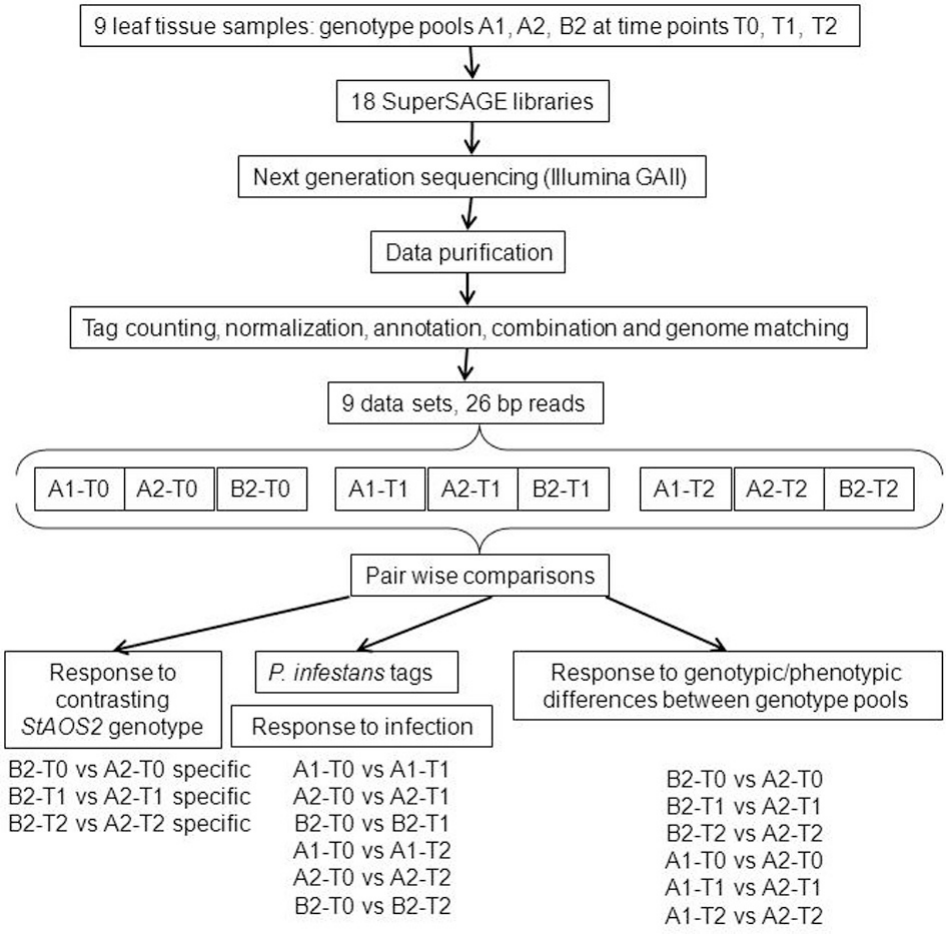
**Flow-chart of SuperSAGE experimental design and data analysis**. The pair wise comparisons used to select differential transcripts according to different rationales (Results sections *P. infestans* tags for monitoring infection, Candidate genes for different MCR levels resulting from the contrasting genotype at the *StAOS2* locus, Potato transcripts differentially regulated upon infection with compatible *P. infestans* isolates, and Potato transcripts differential between genotype pool A2 vs. both pools A1 and B2) are shown at the bottom.

The plants in genotype groups A1, A2, and B2 and their average MCR values provided the basis for comparative transcript profiling by SuperSAGE. Figure 1 shows a flow chart of the experimental design and the data analysis. Nine leaf tissue samples were prepared from plants artificially inoculated with *P. infestans* in a growth chamber. In order to average transcript frequencies across individual, heterozygous genetic backgrounds, SuperSAGE libraries were constructed from total leaf RNA of the pooled 14, 6, and 9 genotypes in groups A1, A2, and B2, respectively, prior to infection (T0), one (T1), and 2 days (T2) after infection (see Materials and Methods). The combined tag counts of both *Nla*III and *Dpn*II libraries resulted in 1.1 to 6.2 million tags per sample (Supplementary Table S1). Of total 266,361 unique tags (unitags), 52.6% mapped to the potato genome sequence (Xu et al., 2011) when allowing up to three mismatches per 26 base pairs, and 23.3% mapped without mismatch. Allowing three mismatches took into account the high level of intra-specific DNA polymorphism in *Solanum tuberosum* Group *tuberosum* genotypes (Rickert et al., 2003) and the fact that tags from *S. tuberosum* Group *tuberosum* genotypes were matched against the genome of a related, homozygous diploid genotype “DM” of *Solanum phureja* Group *tuberosum* (Xu et al., 2011). Moreover, allowing mismatches enables the detection of allelic tags (two slightly different tags matching to the same position in the genome). 67.7% and 73.5% of the unitags with up to three and zero mismatches, respectively, were located in annotated genes, while the rest matched to putative intergenic regions (Table 3, Supplementary Tables S2, S3).

**Table 3:** **Summary of tags matching to the potato genome sequence**.

	**Total no. of unitags**	**No. of matching unitags**	**No. of unitags matching to gene**	**No. of unitags matching to putative intergenic regions**
Up to 3 mismatches	266 361	140 046	94 452	45 595
No mismatch	266 361	61 970	45 567	16 403

#### Pair wise comparisons between SuperSAGE samples

Fifteen pair wise comparisons were performed between the nine samples in order to identify transcripts differentially expressed in response to infection (six comparisons: three genotype pools, two time points) or between the three genotype pools before (T0), one (T1), and 2 days (T2) after infection (nine comparisons: three genotype pools, three time points). The number of unitags per comparison ranged from 127,000 to 182,000 (Supplementary Table S4). When matched against expressed sequence databases of potato (DFCI potato gene index) and *P. infestans* (Materials and Methods), on average 57% of the unitags did not match any expressed sequence (“no hits”). This was in part due to tags consisting mainly of A stretches derived from polyadenylated RNA. The occurrence of non-matching tags without obvious A stretches indicated that a portion of transcribed potato genes was not represented in the DFCI database. On the other hand, many tags matched to more than one expressed sequence (transcript) with similar annotation (see Supplementary Tables S8 and S10 below). In most cases, these transcripts matched with the top score to the same locus in the potato genome, suggesting that they corresponded to allelic variants. Ambiguities arose when transcripts matched with similar high scores to different members of multi-gene families or to transcripts with inconsistent annotations (see below).

The numbers of differentially expressed tags obtained in the pair wise comparisons when using arbitrary cut-off values of *p* < 10^−6^, *p* < 10^−5^ and *p* < 10^−4^ for the likelihood of a difference, are summarized in Supplementary Table S4. For subsequent analyses the cut-off value *p* < 10^−4^ was used throughout. With this threshold, an average of 5% (between 3207 and 11,771) of all tags in a sample showed differential expression, two orders of magnitude more than expected by chance alone. The highest number of differences was observed for the comparison between genotype pools A1 and A2 one day after infection (A1-T1 vs. A2-T1), and the lowest between genotype pools A2 and B2 2 days after infection (B2-T2 vs. A2-T2) (Supplementary Table S4). The number of differences in response to infection and between genotype pools even before infection (T0), was in the same order of magnitude. In all comparisons, approximately one half of the significant tags was differentially regulated in the opposite direction to the other half (up and down compared to T0, higher in pool A compared to B and vice versa).

**Figure 2:**
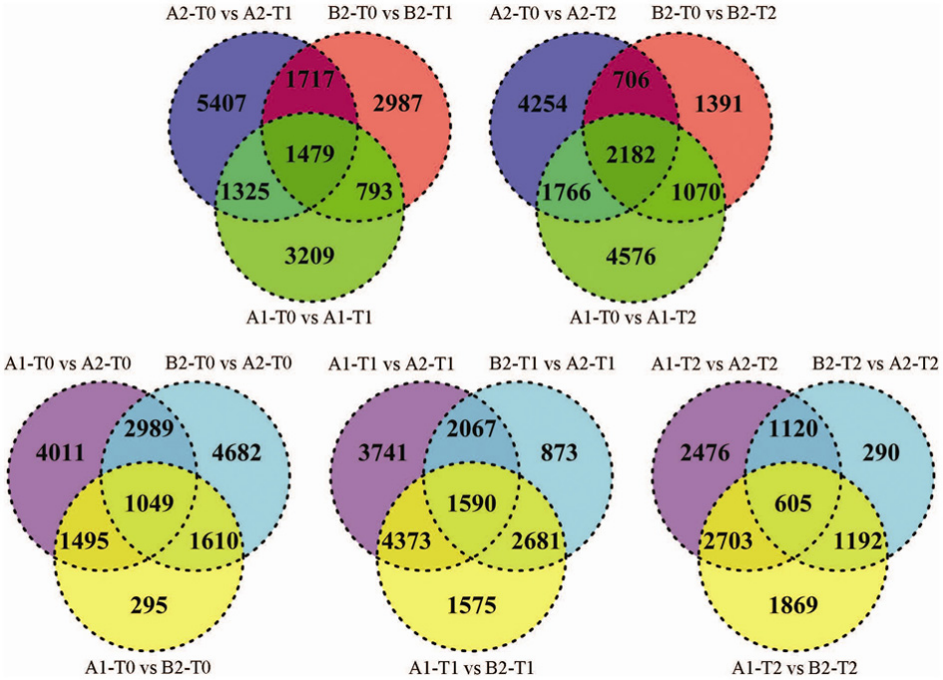
**Venn-diagrams for number of significant differences (*p* < 10^−4^) between tag frequencies in pair wise comparisons**. Upper row: comparison of genotype pools A1, A2, and B2 at T0 vs. T1 (left) and T2 (right). Lower row: comparison between genotype pools A1 vs. A2, B2 vs. A2 and A1 vs. B2 at T0 (left), T1 (middle), and T2 (right).

Forty three percent of the tags (average 67,800) matched to approximately 30,000 transcripts per sample, of which an average of 11% were differentially expressed (Supplementary Table S4). Venn-diagrams of significant tags in the comparisons over the infection time course and between genotype pools (Figure 2) showed that up to 50% of the differential tags were comparison specific. This category of tags likely included the largest portion of false positive differential tags due to random biological variation of the leaf transcriptome. On the other hand, the 2476 (T2), 3741 (T1), and 4011(T0) differential tags specific for the comparisons between pools A1 and A2 included tags differentially expressed due to the fact that pools A1 and A2 originated from different crosses and/or differed in MCR values. Among the 290 (T2), 873 (T1), and 4682 (T0) differential tags specific for the comparisons between pools A2 and B2 should be those tags that are the consequence of the contrasting genotype at the *StAOS2* locus and/or the different MCR levels. Between 600 and 4400 tags were differentially expressed in two or three comparisons. Tags differentially expressed in multiple comparisons are more likely to be functionally significant. We therefore, combined pair wise comparisons according to various rationales (Figure 1 and below), in order to filter the data for transcripts that possibly play a functional role in the compatible interaction with *P. infestans* and in MCR.

#### P. infestans tags for monitoring infection

**Figure 3:**
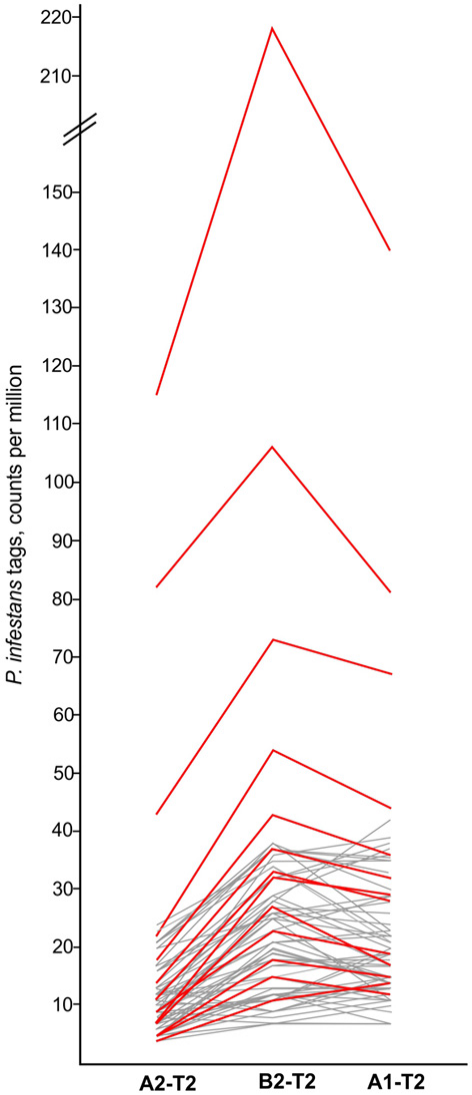
**Normalized tag counts (tpm) of 66 *P. infestans* tags up regulated in genotype pools A1, A2, and B2 2 days after infection (T2)**. A subset of tags showing typical expression patterns are highlighted red. For detailed tag counts see Supplementary Table S5.

The six comparisons of pools A1, A2, and B2 before (T0) and after infection (T1, T2) (Figure 1) were filtered for tags matching *P. infestans* transcripts, which were absent at T0 and induced upon infection in all three genotype pools. Tags matching to *P. infestans* transcripts, which were already present at T0 were not considered authentic. They likely corresponded to transcripts of potato or non-pathogenic microorganisms colonizing potato leaves, which share high sequence similarity with transcripts of *P. infestans*. At T1 the frequency of *P. infestans* tags was negligible (not shown). At T2 sixty six tags fulfilled the selection criteria (Supplementary Table S5). The majority of these tags matched *P. infestans* ribosomal genes and other components of the translation machinery (translation elongation factor 1-alpha, nascent polypeptide-associated complex subunit alpha-like). The second most frequent class corresponded to hypothetical proteins. Among the rest, tags matching to the elicitors NPP1 (necrosis-inducing *Phytophthora* protein 1 (Kanneganti et al., 2006) (tag 9 in Supplementary Table S5), surface glycoprotein elicitor (Kamoun et al., 1997) (tag 63 in Supplementary Table S5) and elicitin-like INF7 (Kamoun et al., 1999) (tag 27 in Supplementary Table S5) were observed. Most importantly, the normalized tag counts (tpm, tags per million) of 90% of the *P. infestans* tags were lower in pool A2 as compared to both pools A1 and B2 (Figure 3, Supplementary Table S5), consistent with the resistance ranking of pools A1, A2, and B2, with pool A2 being the most resistant (Table 1).

#### Differential expression of alleles at the StAOS2 locus

**Figure 4:**
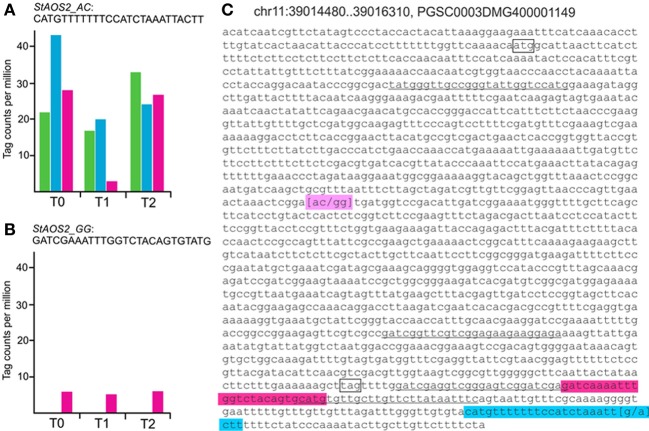
**Tags derived from the *StAOS2* locus**. Normalized tag counts (tpm) of two tags corresponding to haplotypes *StAOS2_A*_*691*_*C*_*692*_ **(A)** and *StAOS2_G*_*691*_*G*_*692*_ **(B)** in genotype pools A1 (green), A2 (blue) and B2 (pink) before (T0), one (T1) and 2 days (T2) after infection with a compatible isolate of *P. infestans*. For detailed tag counts see Supplementary Table S6. **(C)** Genomic sequence of *StAOS2* (PGSC0003DMG400001149). Start and stop codon are shown in boxes. Four secondary tag sequences are underlined. The primary tags corresponding to haplotypes *StAOS2_G*_*691*_*G*_*692*_ and *StAOS2_A*_*691*_*C*_*692*_ are highlighted pink and blue, respectively. The position of SNPs 691 and 692 is highlighted magenta.

We tested whether SuperSAGE tags corresponding to the two *StAOS2* haplotypes used for constructing the genotype pools A1, A2, and B2 were detectable and whether expression differences could be observed. Filtering Supplementary Table S2 for tags matching to the *StAOS2* locus on potato chromosome XI (PGSC0003DMG400001149) yielded seven tags, four of which originated from secondary restriction sites, resulting from incomplete digestion of the primary restriction site upstream of the poly(A) tail during SAGE library construction (Figure 4C). The frequencies of the secondary tags were very low and no significant differences between samples were detected (not shown). One of the three tags derived from primary restriction sites (Figure 4, Supplementary Table S6) was detected at low frequency exclusively in pool B2 (Figure 4B). This tag therefore, corresponded to haplotype *StAOS2_G*_*691*_*G*_*692*_, which was present in triple dosage in B2 plants but absent in A1 and A2 plants. The second and third tag were allelic variants that differed by only one nucleotide at position 23 (Supplementary Table S6, Figure 4C). The tag with the highest frequency was present in all three genotype pools as was the haplotype *StAOS2_A*_*691*_*C*_*692*_ (Figure 4A), whereas the other was inconsistently detected at very low frequency (tpm < 3) only in pools A1 and B2 (Supplementary Table S6). The frequency of the tag corresponding to haplotype *StAOS2_A*_*691*_*C*_*692*_ was significantly higher in pool A2 as compared to both pools A1 and B2 prior to infection (T0), consistent with the observed MCR ranking of the three pools. At T1 the frequency was significantly lower in pool B2 compared with both pools A1 and A2, corresponding to the expected ranking of the three pools according to the *StAOS2* genotype. At T2 the differences between genotype pools had diminished (Figure 4A, Supplementary Table S6). The expression patterns of these tags demonstrated that (i) SuperSAGE was able to detect the expression of different alleles of the same locus and (ii) the two *StAOS2* haplotypes used for MAS were indeed differentially expressed prior to infection and one day after infection.

#### Candidate genes for different MCR levels resulting from the contrasting genotype at the StAOS2 locus

Tags differentially expressed specifically between pools A2 and B2 but not between pools A2 and A1 can result from the pool's contrasting genotypes at the *StAOS2* locus and/or the different MCR values. The differential tags specific for the three comparisons between pools A2 and B2 at T0, T1 and T2 (Figure 1) were filtered for tags differentially expressed at two or three time points. This resulted in 93 tags, mostly with rather low expression levels (Supplementary Table S7), of which 25 could not be annotated, neither by matching to expressed sequences nor to annotated genes in the potato genome sequence nor by comparing matching transcripts to the NCBI nucleotide collection. Nine tags had ambiguous annotations. Eighty five of the 93 tags were differentially expressed prior to infection, indicating that the genotypic differences between pools A2 and B2 led to constitutive changes in the leaf transcriptome. Thirty eight tags were expressed at higher level in the more resistant A2 pool compared to the more susceptible B2 pool. Among those were putative components of hormone signaling pathways, for example “salicylic acid-binding protein 2″ (SABP2, TC201627) (Kumar and Klessig, 2003), the polypeptide hormone “rapid alkalinization factor 2″ (RALF2, TC205054) (Ryan et al., 2002), “protein phosphatase 2C″ regulating ABA signaling (PP2C, TC212535) (Hubbard et al., 2010), proteins located in chloroplasts, for example “fibrillin 8″ (TC207935) (Singh and McNellis, 2011) and protein “chloroplast import apparatus 2″ (CIA2, PGSC0003DMG400022983, tag 15 in Supplementary Table S7) (Sun et al., 2009), and of cellular redox homeostasis such as “glutaredoxin-C6” (Grx, TC214704) (Meyer et al., 2009). Thirty six tags were expressed at higher level in the B2 pool. These included “chloroplast epoxide hydrolase” (TC218915) functional in lipid metabolism or signaling (Guo et al., 1998), stay green protein (TC206798) involved in senescence (Hörtensteiner, 2009) and tag 26 (Supplementary Table S7) matching to the locus PGSC0003DMG400015546 annotated in the potato genome as “Endoplasmic reticulum-type Calcium ATPase” (Wimmers et al., 1992). This tag was present at a very low level in pool B2 at all three time points but was completely absent in pool A2. The remaining 19 tags showed expression with inverted direction between time points, for example the tag matching TC208517 [putative plastid-lipid-associated protein (Langenkämper et al., 2001)]. Expression of this transcript strongly decreased from T0 to T1 in pool A2 but increased in B2.

#### Potato transcripts differentially regulated upon infection with compatible P. infestans isolates

Up and down regulation of transcripts in response to pathogen attack has been widely studied in plant host-pathogen interactions including potato. Filtering the SuperSAGE tags for such transcripts should therefore, expose the known up regulation of pathogenesis-related (PR) transcripts that could serve as internal controls for an activated defense response. In addition, the dynamic range and depth of digital transcript profiling should enable the discovery of novel defense related genes. The genotype pools A1, A2, and B2 represented the allelic diversity of 14, 6, and 9 half-sib (A1, A2) and full-sib (A2, B2) individuals, respectively, which originated from three heterozygous, tetraploid parental genotypes representing 12 genome wide potato haplotypes. To select transcripts up or down regulated in response to infection with *P. infestans* irrespective of genetic background, the comparisons over the infection time course (Figure 1) were filtered for potato transcripts that fulfilled the following criteria: the normalized hit counts (hpm, hits per million; hit counts are the sum of the counts of all tags matching to the same transcript) showed a difference between T0 and T1 and/or between T0 and T2 in the same direction (up or down) in all three genotype pools. Hit counts instead of tag counts were used to reduce the redundancy in the expression data, as in most cases two or more tags matched to the same transcript. Consistent up or down regulation in three different genotype pools represented a stringent criterion for reproducibility. The tags that did not match to any transcript present in the SGTI expressed sequences database (“no hits”) were filtered with the same criteria. The filtering resulted in 1536 potato transcripts (Supplementary Table S8) and 498 “no hit” tags (Supplementary Table S9), which were differentially expressed upon infection with compatible *P. infestans* isolates. These total 2034 expressed sequences were transcribed from approximately 1830 potato genes, of which 6% were not annotated and 12% were not identified in the potato genome sequence. Tags matching known PR genes such as PR1b, PR10, beta-1,3 glucanases (PR2), several chitinases (e.g., PR3), and thaumatin-like genes (PR5) (Van Loon et al., 2006) were strongly induced (Supplementary Table S8). Two hundred and seventy six “no hit” tags (55%) matched to the potato genome sequence (allowing for three mismatches), 80 of those to intergenic regions. Closer inspection of these intergenic regions in the potato genome revealed that 48 such tags either corresponded to non-annotated genes, as indicated by expressed sequences matching to the corresponding genomic region (genome browser at http://solanaceae.plantbiology.msu.edu/pgsc_download.shtml) or were located in the 3′-untranslated region of annotated genes. Interestingly, 32 tags remained that appeared truly located in intergenic regions. Most of these were up regulated upon infection with *P. infestans* (Table 4, Supplementary Table S9). Twenty five percent of all differentially regulated transcripts had no meaningful functional annotation, and 4.6% had ambiguous annotations, meaning that the tags matched several transcripts with different annotations or transcripts with inconsistent annotation in the SGTI expressed sequences database and the matching locus in the potato genome (Table 4).

**Table 4:** **Grouping of differential transcripts according to putative function category**.

**Function category^a^**	**No. of potato differential transcripts after infection in genotype pools A1, A2, and B2^b^**	**No. of potato differential transcripts between genotype pools A1, A2, and B2^c^**
**Unknown** function	505 (332 ↑, 173 ↓)	166 (16)
Biotic/abiotic/oxidative **stress**, pathogenesis, defense	289 (207 ↑, 82 ↓)	91 (14)
**Chloroplast** processes, photosynthesis, carbon fixation	190 (21↑, 169 ↓)	107 (24)
**Membrane** and **transport** proteins	137 (91 ↑, 46 ↓)	36 (8)
**Transcription**	90 (63 ↑, 27 ↓)	27 (4)
**Protein biosynthesis**	72 (37 ↑, 35 ↓)	84 (9)
**Protein degradation**	68 (54 ↑,14 ↓)	33 (5)
Protein conformation, **chaperone**	42 (27 ↑, 15 ↓)	19 (2)
**Carbohydrate** metabolism	64 (37 ↑, 27 ↓)	10 (1)
**Secondary** metabolism	59 (27 ↑, 32 ↓)	10 (0)
**Cell wall**	57 (23 ↑, 34 ↓)	24 (2)
**Development**, **regulation**, signaling	58 (34 ↑, 24 ↓)	15 (0)
**Lipid** metabolism	54 (34 ↑, 20 ↓)	14 (2)
**Central metabolism**	48 (29 ↑, 19 ↓)	29 (3)
**Hormone** metabolism/signaling	44 (27 ↑, 17 ↓)	14 (3)
**Amino acid metabolism**	39 (22 ↑, 17 ↓)	15 (3)
**Other**	257 (171 ↑, 86 ↓)	92 (15)
**Intergenic** regions	32 (25 ↑, 7 ↓)	14 (1)
**No match** in potato genome	226 (141 ↑, 85 ↓)	88 (6)
**Ambiguous** annotation	93 (44 ↑, 49 ↓)	67 (9)
Total	2034 (1237 ↑, 797 ↓)	806 (112)

a Words in bold letters can be used to text filter Supplementary Tables S8–S11 in columns “putative function,” “chromosome” (no match) and “locus” (intergenic).

b The number of up (↑) and down (↓) regulated transcripts are shown in parenthesis.

c The number of transcripts showing the MCR conform expression pattern at all time points are shown in parenthesis.

#### Potato transcripts differential between genotype pool A2 vs. both pools A1 and B2

Comparisons between genotype pools at the same time point were performed in order to identify candidate genes responsible for the different MCR levels of the genotype pools. The rationale for data filtering was as follows: Besides from random biological variation, differential transcript levels between genotype pools A1 and A2 could result from the different MCR levels of these pools as well as from the fact that A1 and A2 genotypes originated from different cross combinations. On the other hand, differential transcript levels between pools B2 and A2 can be the consequence of the contrasting genotype at the *StAOS2* locus and/or the higher MCR in pool A2 as compared to B2 (see above). Transcripts expressed at higher or lower level in pool A2 as compared to both pools A1 and B2 are therefore, candidates for playing a role in MCR, irrespective of the genotype at the *StAOS2* locus. To identify such transcripts, six comparisons between pool A1 vs. A2, and B2 vs. A2, at the infection time points T0, T1, and T2 (Figure 1) were filtered for differential transcripts. To reduce random differences further, hpm had to be significantly different in at least five of the six comparisons. The “no hit” tags were filtered with the same criteria. Under these conditions, 629 potato transcripts (Supplementary Table S10) and 177 “no hit” tags (Supplementary Table S11) were selected, which were transcribed from approximately 720 genes. Around 19% of these genes were either not annotated or not detected in the potato genome sequence. One half of the “no hit” tags matched to the potato genome sequence, 14 of those to intergenic regions. Twenty percent of all transcripts could not be functionally annotated and 7.3% had ambiguous annotations (Table 4).

In order to prioritize further the total 806 transcripts with respect to their potential relevance for MCR, additional selection criteria were applied. First, the transcripts were examined for an expression pattern corresponding to the observed MCR values, that is higher or lower expression in genotype pool A2 when compared to both pools A1 and B2. Prior to infection (T0), 75% of the transcripts showed this expression pattern, to which we refer subsequently as the “MCR conform expression pattern.” This number dropped to 51% and 54% at T1and T2, respectively. MCR conform expression patterns at all three time points were observed for 112 transcripts (highlighted green in Supplementary Tables S10 and S11). Three hundred and fifty two and 256 transcripts were expressed at higher and lower level, respectively, in uninfected plants (T0) of pool A2 as compared to both pools B2 and A1. Two days after infection (T2), 245 transcripts were expressed at higher level in pool A2 as compared to both pools B2 and A1, and 196 transcripts were expressed at lower level. Expression patterns at T1 were more heterogeneous due to transient up or down regulation of transcripts. Second, 281 transcripts were selected, which showed consistent differential expression after infection with *P. infestans* as well as between genotype pools (Supplementary Table S12).

### Functional categories of differential transcripts

Based on the annotations in the DFCI potato gene index, of the matching loci in the potato genome and in few cases by BLAST searches against the NCBI database (http://blast.ncbi.nlm.nih.gov/Blast.cgi), transcripts that showed consistent differential expression over the infection time course or between genotype pools A1, A2, and B2, were grouped in 17 functional categories, with overlaps between categories (Table 4, Supplementary Tables S8–S11). The largest group of differential tags/transcripts did not allow any function assignment. It included, besides tags derived from annotated and non-annotated genes of unknown function, 226 tags that did not match to the potato genome sequence. Many but not all of these tags contained A stretches originated from the poly(A) tail of mRNA, which prevented genomic matching. Non-matching tags without A stretches may derive from genes not covered by the draft potato genome sequence or from genes highly divergent between *S. tuberosum* Group *phureja* (genome sequence) and *S. tuberosum* Group *tuberosum*, from which the samples originated. The largest groups of transcripts with functional annotation were related to biotic as well as abiotic stress responses and to chloroplastic processes. The majority of transcripts were up regulated upon infection with *P. infestans*, except transcripts functional in the chloroplast, which were mostly down regulated. Details on the individual functional categories are provided as Supplementary material (Supplementary Results 3.1–3.17).

### Co-localization of differentially expressed genes and resistance QTL

**Figure 5:**
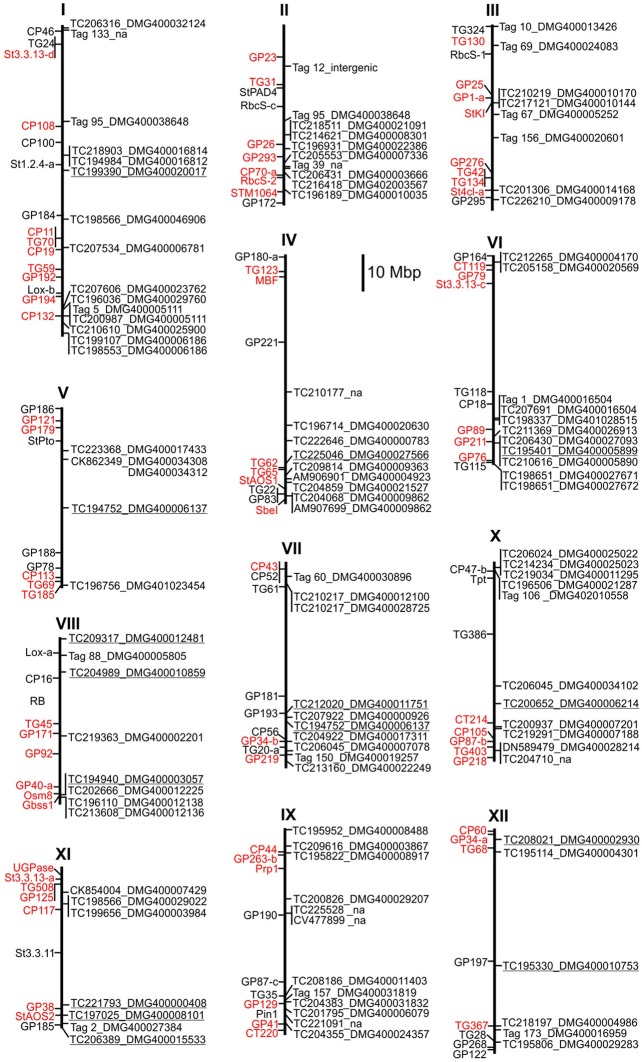
**Physical map of resistance QTL and candidate genes showing the MCR conform expression pattern at all time points or were tested by qRT-PCR**. To the left of each chromosome, RFLP markers linked to resistance QTL (Leonards-Schippers et al., 1994; Zimnoch-Guzowska et al., 2000; Ballvora et al., 2011; Danan et al., 2011) are shown in red and additional chromosome anchor markers in black. To the right of each chromosome, the positions of candidate loci and matching transcripts are shown. Tags not matching to any transcript in the SGTI database (no hits) are numbered according to Supplementary Table S11. Loci analyzed by qRT-PCR are underlined. na = transcript not annotated in the potato genome.

Overlapping genomic positions of candidate genes with resistance QTL can be used as additional criterion to rank a large number of functional candidates. QTL linkage mapping for resistance to late blight and other pathogens of potato has been performed in several genetic backgrounds using as markers restriction fragment length polymorphisms (RFLP's) of potato and tomato (Gebhardt and Valkonen, 2001; Simko et al., 2007; Danan et al., 2011). Resistance QTL were anchored to the potato genome sequence by positioning linked RFLP markers on the physical maps of the 12 potato chromosomes (Xu et al., 2011) (Figure 5). Due to variable ratios between genetic and physical map distances (Xu et al., 2011), sizes and positions of resistance QTL on the physical maps deviated from previous consensus maps for resistance QTL based on genetic linkages (Gebhardt and Valkonen, 2001; Danan et al., 2011) [see also the Solanaceae (SOL) function map for pathogen resistance at http://www.gabipd.org/database/maps.shtml]. The genomic positions of transcripts that showed the MCR conform expression pattern at all time points and matched with high confidence to unique loci are shown on the physical map (Figure 5). Several of these transcripts matched to novel loci, not annotated in the potato genome, and one matched to an intergenic region on chromosome II. Co-localization with resistance QTL was observed for a number of loci. For example, the central QTL for late blight resistance on chromosome III (*Pin3B* and *lb3* on the SOL function map for pathogen resistance at http://www.gabipd.org/database/maps.shtml) extended over approximately 3 Mbp. This region contains the *StKI* locus, which consists of mixed clusters of protease inhibitors genes (Odeny et al., 2010). The inhibitor encoded at the locus PGSC0003DMG400010170 was expressed consistently at higher level in pool A2, whilst the inhibitor encoded at the tightly linked locus PGSC0003DMG400010144 was expresses at lower level in pool A2 compared with both pools A1 and B2 (Figure 5, Supplementary Table S10).

### Semiquantitative real time PCR (qRT-PCR) of selected candidate genes

**Figure 6:**
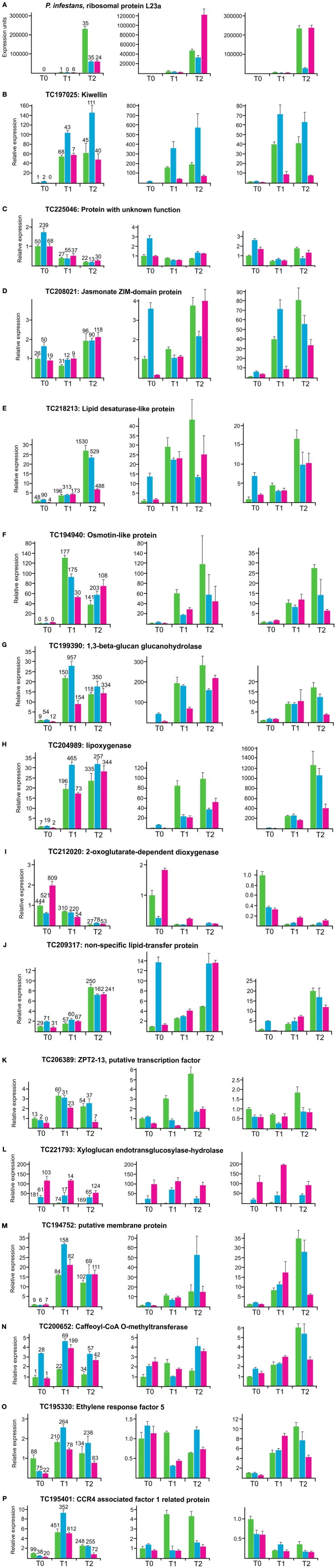
**Expression analysis by qRT-PCR of a *P. infestans* transcript as internal control for successful infection (A), four control genes known to be induced (osmotin-like protein, 1,3-beta-glucan glucanohydrolase, lipoxygenase) or repressed (2-oxoglutarate dependent dioxygenase) upon infection **(F–I)** and eleven novel candidate genes **(B–E, J–P)** in three independent infection experiments**. The left panels show the results when using RNA of the same plant tissues as used to construct the SuperSAGE libraries. Relative expression in genotype pools A1, A2, and B2 is shown as green, blue, and pink bars, respectively. The normalized hit counts of each transcript are indicated on top of each bar (numbers rounded). The middle and right panels show the results obtained in experiment two and three. Error bars were derived from two technical replicates. They represent the maximum deviation from the mean.

qRT-PCR was performed with one *P. infestans* transcript and 15 potato transcripts, in order to assess the reproducibility of differential expression in independent infection experiments. (Figure 6). The genomic positions of the corresponding potato loci are included in Figure 5. The transcript for the ribosomal protein L23a of *P. infestans* (Figure 6A) and potato transcripts known to be up (osmotin, 1,3-beta-glucan glucanohydrolase, lipoxygenase) (Ros et al., 2004; Restrepo et al., 2005; Gyetvai et al., 2012) or down regulated (2-oxoglutarate-dependent dioxygenase) (Restrepo et al., 2005) in response to infection by a compatible *P. infestans* isolate served as controls (Figures 6F–I). The remaining transcripts were chosen based on novelty according to their annotation, strong differential expression upon infection (log-fold change = 2) and differences between genotype pools. The strong increase in *P. infestans* L23a transcripts after 2 days confirmed successful infection in the three experiments analyzed (Figure 6A). Up or down regulation as quantified by normalized hit counts (hpm) in SuperSAGE was reproducible for all transcripts tested in cDNA's of experiment 1, the same as used for generating the SuperSAGE samples. High relative expression levels in qRT-PCR did however, not correspond to high hpm counts in SuperSAGE and vice versa, indicating that both methods for quantifying transcript levels were qualitative but not quantitative comparable. Also the relative ranking of the three genotype pools based on hpm at the three time points was in 40% of the cases not congruent with the ranking observed in qRT-PCR. A particularly intriguing example was transcript TC221793 (Supplementary Table 10), which had the highest hit counts in genotype pool A1 compared with pools A2 and B2, but was not detectable by qRT-PCR in pool A1, whereas the lower transcript level in pool A2 vs. B2 was observed in all three experiments (Figure 6L). In experiment 2 and 3, induction or repression was generally reproducible, except for transcripts TC195330 and TC195401, which showed inconsistent results in one of three experiments (Figures 6O,P). As observed previously (Gyetvai et al., 2012), relative expression levels and time courses were highly variable between experiments. The most reproducible expression pattern showed transcript TC197025 (Figure 6B) annotated as “kiwellin,” which was originally described as allergen from Kiwi fruits (Tamburrini et al., 2005). This transcript was strongly up regulated after infection in all genotype pools and raised to higher levels in pool A2 compared to both pools A1 and B2. The MCR conform expression pattern was also reproducibly detected at T0 for transcripts TC225046 (Figure 6C), TC208021 (Figure 6D), TC218213 (Figure 6E) and TC209317 (Figure 6J).

## Discussion

### MAS for maturity corrected resistance to late blight

To the best of our knowledge, the MAS experiment described here is the first where, based on diagnostic SNP markers, plants with improved quantitative resistance to late blight not compromised by late maturity were selected. The results show that the predictive power of the two *StAOS2* SNP markers depends on the genetic background, in which MAS is performed. Genotypic selection in two half-sib families based on two *StAOS2* haplotypes, one expected to have positive and the other negative effects on MCR, resulted in genotype groups A1, A2, and B2. Groups A1 and A2 were homozygous for the positive *StAOS2* haplotype, whereas group B2 carried one copy of the positive and three copies of the negative haplotype. As expected, group A2 was more resistant than group B2. Unexpected, group A1 was as susceptible as group B2 (Table 1). The reason for the observed difference in MCR level between groups A1 and A2 may be the missing genotypic information at additional, unknown loci that also influence MCR. The two SNPs at the *StAOS2* locus explained approximately one third of the genetic variation of MCR (Pajerowska-Mukhtar et al., 2009), which means that two thirds of the genetic variation were due to other loci. The alleles present at these additional loci might have been different in parents Phy14 and Phy16, thereby modifying or even suppressing the effects of *StAOS2* alleles on MCR depending on the genomic context. Moreover the haplotypes *StAOS2_A*_691_*C*_692_ and *StAOS2_G*_691_*G*_692_ are not uniform with respect to additional SNPs at the *StAOS2* locus. This was indicated by finding two allelic tags corresponding to haplotype *StAOS2_A*_691_*C*_692_ (Results Section Differential expression of alleles at the *StAOS2* locus). Multiple, segregating *StAOS2* alleles may modulate the phenotypic effects of the specific SNPs used for MAS.

### *P. infestans* transcripts diagnostic for quantitative disease progression

Tags derived from *P. infestans* transcripts allowed to monitor the infection and disease progression during the initial, biotrophic phase of the compatible interaction, when no disease symptoms were visible. They provided a convenient internal infection control. *P. infestans* transcripts strongly increased 2 days after infection. In contrast, *P. infestans* DNA is detected and quantified *in planta* by species-specific PCR only 3 to 4 days after infection (Judelson and Tooley, 2000). Moreover, the frequency of 90% of *P. infestans* tags 2 days after infection was smallest in the most resistant pool A2 and thereby corresponded to the ranking of the genotype pools A1, A2, and B2 according to MCR. This suggests that the molecular processes occurring during the first 2 days after artificial inoculation in a growth chamber mimic in a time lapse the progression of a late blight infestation in the field, at least to some extent.

### Differential expression of *StAOS2* alleles and putative jasmonate responsive transcripts

The sensitivity of digital transcript profiling by SuperSAGE was demonstrated by the differential tags corresponding to the *StAOS2* alleles that were used to select A1, A2, and B2 plants. The negative *StAOS2_G*_691_*G*_692_ haplotype present only in B2 plants was expressed at much lower level than the positive haplotype *StAOS2_A*_691_*C*_692_. For both haplotypes no differential regulation in response to infection was observed. Moreover, *StAOS2_ A*_691_*C*_692_ showed the MCR conform expression pattern in uninfected plants (Figure 4). This expression pattern suggests that StAOS2 transcript levels are a limiting factor for the production of jasmonates (Kombrink, 2012). Higher StAOS2 transcript levels in uninfected plants may lead to higher constitutive amounts of one or more products of the jasmonate pathway (Göbel et al., 2002), consequently to increased jasmonate signaling and to higher constitutive expression of jasmonate responsive genes (Reymond and Farmer, 1998), which could influence quantitative pathogen resistance. Indeed, higher transcript levels prior to infection in pool A2 as compared to pools B2 and A1 were observed for known jasmonate responsive genes such as lipoxygenases (TC196405, TC204989) and chitinases (TC194776, TC198089, TC220862, TC222828, Supplementary Table S10). This model is consistent with functional analyses of potato and rice AOS genes in transgenic plants. AOS silencing led to increased susceptibility and AOS overexpression to increased resistance (Mei et al., 2006; Pajerowska-Mukhtar et al., 2008). Some of the differential transcripts specific for the comparisons between genotype pools A2 and B2 (Supplementary Table S7) might be components of a regulatory network in which StAOS2 participates, particularly those that show, like *StAOS2*, differential expression prior to infection. This set of transcripts corresponded mostly to genes of unknown function or with very unspecific annotation, most of them expressed at low levels. Annotated genes had a putative function in hormone, lipid, and calcium signaling, proteasome mediated protein degradation, transport and in chloroplastic processes, for example fibrillin 8 (TC207935, Supplementary Table S7). Fibrillin 8 may have a role in that part of jasmonate biosynthesis which takes place in the chloroplast (Singh and McNellis, 2011). The same transcript was down regulated in compatible interactions of potato with *P. infestans* based on transcript profiling by DeepSAGE (Gyetvai et al., 2012). The expression patterns of the tags matching to fibrillin 8 were similar in both transcript profiling experiments.

### Differential expression upon infection with compatible isolates of *P. infestans*

The comparisons of infected with non-infected plants in genotype pools A1, A2, and B2 resulted in approximately 2000 potato transcripts, 25% with unknown function, that were either up or down regulated during the compatible interaction with *P. infestans* (Table 4). The transcripts mapped to approximately 1830 loci in the potato genome, since a fraction of the transcripts corresponded to alleles of the same locus. Induction or repression upon infection was confirmed by qRT-PCR for most of the 15 genes tested, although infection kinetics varied substantially between independent infection experiments. Validity of our sampling strategy was demonstrated by observing the expected expression patterns of well known pathogenesis related genes, which served as internal controls besides *P. infestans* transcripts. The compatible interaction activated not only responses to biotic but also to abiotic stress, indicating that the distinction between biotic and abiotic stress is rather artificial and results from the way scientists study plant-environment interactions rather than from naturally distinct response pathways. The differential expression of components of most known hormone signaling pathways also supports the view that plants react to a compatible *P. infestans* infection with a global stress response. A clear symptom of senescence and beginning cell death was the down regulation of numerous transcripts for chloroplast processes, particularly for photosynthesis and chloroplastic protein biosynthesis, which was observed already 1 day after infection. Down regulation of photosynthesis has been observed in many transcriptome studies and is an unspecific plant reaction to pathogen attack (Bilgin et al., 2010).

### Differential expression between genotype pools with different MCR levels

The 806 transcripts from approximately 720 genes, which showed most consistent differential expression between the genotype pools A1, A2, and B2 are considered as primary candidates for playing a functional role in MCR (Supplementary Tables S10, S11). This type of differential expression has not been analyzed before. Top candidates among those might be the genes that showed the MCR conform expression pattern before as well as after infection with *P. infestans*, and the genes that were induced or repressed upon infection in addition to showing differences between genotype pools (Supplementary Table 12). However, the 806 candidate genes include an unknown portion of false positives. Transcript levels differing between genotype pools were less reproducible in independent infection experiments than transcript induction or repression upon infection, as assessed by qRT-PCR for a small number of candidates. Best reproducibility was achieved for differences present in uninfected plants, likely due to the variability of *P. infestans* infection kinetics. On the other hand, sensitivity and resolution of qRT-PCR might be insufficient for validation of quantitative differences between low expressed, sometimes allele specific transcripts detected by SuperSAGE.

Differential expression originates either directly or indirectly from DNA variation in genes causal for MCR. The former possibility has to be distinguished from the latter when aiming at the identification of diagnostic markers for breeding applications. Diagnostic DNA markers have to reside within the genes causal for MCR or be in linkage disequilibrium with the causal genes. The identification of such markers requires association mapping, as exemplified for *StAOS2* (Pajerowska-Mukhtar et al., 2009). The genes showing differential expression between genotype pools A1, A2, and B2 set the stage for the discovery of additional diagnostic markers for MCR and of genes underlying quantitative resistance to late blight. The confirmation of a function in pathogen resistance of individual candidate genes requires further studies such as gene silencing and complementation analysis of corresponding mutants in the model plant Arabidopsis (Pajerowska-Mukhtar et al., 2008). Such studies can be practically performed only with a small number of genes. The physical map for resistance QTL (Figure 5) provides an auxiliary tool for choosing the most interesting candidates for further analysis, that is the co-localization of candidate gene and resistance QTL. The criterion of co-localization is not exclusive, as QTL delimitations are not precise and not all potato QTL are known. Whole genome association mapping (Zhu et al., 2008) using genome wide SNP's (Hamilton et al., 2011) will be another option for choosing candidates based on co-localization with peaks of marker-trait associations.

The transcripts differentially expressed between genotype pools A1, A2, and B2 were only partially identical with the transcripts differentially expressed upon infection (Table 4, Supplementary Table S12). This shows that MCR is not only controlled by genes induced or repressed upon infection but also by constitutively expressed genes. The majority of the 806 transcripts showed the MCR conform expression pattern prior to infection, that is higher or lower expression in genotype pool A2 compared to both pools A1 and B2. This also indicates that part of the quantitative resistance to late blight is constitutive. Genes with genotype dependent, constitutive differential expression provide excellent targets for developing novel diagnostic tools for breeding cultivars with improved quantitative resistance to late blight and possibly other biotic and abiotic stresses. Approximately 200 of 266,361 unitags matched to 90 *R* genes or *R* gene homologs, of which only two were differentially expressed between genotype pools, based on our stringent filtering criteria. Considering that the potato genome contains 438 putative *R* genes (Jupe et al., 2012), most of these genes were not expressed in leaves before and after infection, or their expression was below detection level. This class of genes does not seem to play a dominant role in MCR, at least not at the transcript level. Genes with roles in downstream defense signaling and defense responses were present more frequently among the 806 candidates. In addition to these, our comparative transcript profiling experiment identified novel candidate genes for MCR in general cellular processes such as photosynthesis, protein biosynthesis, protein degradation via the 26S proteasome, transport of proteins, lipids, ions and other small molecules, in structural cellular components such as the cell wall and the cytoskeleton. Resistance to late blight not compromised by late maturity might be associated with a generally more “healthy” physiological state of the plant, which involves most aspects of metabolism. The MCR conform expression pattern was also observed for numerous genes of unknown or ill-defined function. One such gene was a potato homolog of the allergen “kiwellin” in Kiwi fruits (Tamburrini et al., 2005; Ciardiello et al., 2008; Tuppo et al., 2008). The expression of this gene was more strongly up regulated upon infection in the A2 pool than in both the A1 and B2 pools in three infection experiments (Figure 6B). This expression pattern makes it a potential transcriptional marker for MCR. The MCR conform expression pattern was also observed for some tags matching to intergenic regions. The sequences in these regions require further analysis. They might encode microRNA precursor transcripts (Cuperus et al., 2011; Li et al., 2012) or novel genes, which are neither annotated in the potato genome nor represented in expressed sequence databases.

## Conclusions

1. Based on diagnostic SNPs in the *StAOS2* gene, it was possible to select potato plants with increased resistance to late blight not compromised by late maturity. Additional diagnostic markers are required to improve predictability of MCR independent from genetic background.
2. Comparing by SuperSAGE the expression profiles of genotype pools before and after infection with *P. infestans* yielded a large number of novel candidate genes for quantitative resistance to late blight. They constitute a rich resource for marker development in breeding applications as well as for functional studies, which may unravel molecular mechanisms governing quantitative plant pathogen resistance.
3. Generally, induction or repression of selected transcripts upon infection with *P. infestans* was reproducible by qRT-PCR in three independent infection experiments. Differential expression between genotype pools was in part reproducible by qRT-PCR, particularly differences prior to infection.
4. Comparative expression profiling by SuperSAGE revealed quantitative differences between *StAOS2* alleles prior to infection, which suggest a mechanism for different resistance levels caused by natural variation at the *StAOS2* locus.
5. Maturity-corrected resistance to late blight is, in part, constitutive. This was shown by detecting numerous differential transcripts in uninfected plants showing different levels of MCR.
6. Maturity-corrected resistance to late blight involves, besides biotic and abiotic stress responses mediated by hormone signaling, cellular housekeeping processes like photosynthesis, protein biosynthesis and degradation, transport of proteins, lipids, ions and other small molecules.

### Conflict of interest statement

The authors declare that the research was conducted in the absence of any commercial or financial relationships that could be construed as a potential conflict of interest.

## Supplementary material

Supplementary Table S1 **Number of tags per sample. (Sheet 1)**.

Supplementary Table S2 **Tags matching to the potato genome with maximum 3 mismatches. (Sheet 2)**.

Supplementary Table S3 **Tags matching to the potato genome with no mismatch. (Sheet 3)**.

Supplementary Table S4 **Summary of differential tags/transcripts in 15 pair wise comparisons. (Sheet 4)**.

Supplementary Table S5 ***Phytophthora infestans* tags. (Sheet 5)**.

Supplementary Table S6 ***StAOS2* tags. (Sheet 6)**.

Supplementary Table S7 **Tag counts different specifically between pools A2 and B2.** Higher expression in pool A2 vs. B2 is highlighted green in column J, opposite expression differences at different time points are highlighted orange. (Sheet 7).

Supplementary Table S8 **Transcripts differential in response to infection with *P. infestans*.** Down regulated transcripts are highlighted yellow in columns K and L. (Sheet 8).

Supplementary Table S9 **“No hit” tags differential in response to infection with *P. infestans*.** Down regulated tags are highlighted yellow in columns K and L. (Sheet 9).

Supplementary Table S10 **Transcripts differential between genotype pools.** Transcripts showing the MCR conform expression pattern at all time points are highlighted green in columns M to R. (Sheet 10).

Supplementary Table S11 **“No hit” tags differential between genotype pools.** Tags showing the MCR conform expression pattern at all time points are highlighted green in columns M to R. (Sheet 11).

Supplementary Table S12 **Transcripts and “no hit” tags differential in response to infection and between genotype pools. (Sheet 12)**.

Supplementary Results 3.1–3.17 **Description of functional categories of differential transcripts. (Sheet 13)**.
