# Supplementary material for: Comparative transcript profiling by SuperSAGE identifies novel candidate genes for controlling potato quantitative resistance to late blight not compromised by late maturity
Source: Front Plant Sci. 2013 Nov 14;4:423. doi: 10.3389/fpls.2013.00423 (PMC3827546; doi:10.3389/fpls.2013.00423)
Supplement: Supplementary Table S1 — Number of tags per sample. (Sheet 1). [file DataSheet1.DOCX]

**Supplementary Table 1: Number of tags per sample**

| Sample | No 26 bp TAGs |
| --- | --- |
| A1-T0 | 1 717 726 |
| A2-T0 | 6 187 411 |
| B2-T0 | 1 491 358 |
| A1-T1 | 4 087 751 |
| A2-T1 | 3 297 517 |
| B2-T1 | 1 143 084 |
| A1-T2 | 3 849 376 |
| A2-T2 | 2 078 745 |
| B2-T2 | 1 608 353 |
|  | ∑= 25 461 321 |
