# Supplementary material for: Comparative transcript profiling by SuperSAGE identifies novel candidate genes for controlling potato quantitative resistance to late blight not compromised by late maturity
Source: Front Plant Sci. 2013 Nov 14;4:423. doi: 10.3389/fpls.2013.00423 (PMC3827546; doi:10.3389/fpls.2013.00423)
Supplement: Supplementary Results 3.1–3.17 — Description of functional categories of differential transcripts. (Sheet 13). [file DataSheet13.DOCX]

**Description of functional categories of differential transcripts.**

**3.1.Unknown function.** Transcripts for which no putative function was found by sequence comparisons comprised the largest group. Interesting examples with a perfect MCR conform expression pattern were tag numbers 25, 44, 75 and 171 (Supplementary Table S11) and allelic transcripts CV477899 and TC225528, which show complementary expression (Supplementary Table S10). Transcripts strongly up regulated after infection with *P. infestans* such as CK860494, DN589212 and TC214248 (Supplementary Table S10) can be considered as novel pathogenesis related genes ([van Loon et al., 2006](#_ENREF_64)).

**3.2 Biotic, abiotic and oxidative stress, pathogenesis, defense***.* The second largest group were transcripts putatively functional in biotic as well as in abiotic stress responses (Table 3). Transcripts mostly up regulated after infection encoded classical pathogenesis related (PR) proteins ([van Loon et al., 2006](#_ENREF_64)), peroxidases ([Almagro et al., 2009](#_ENREF_1)), glutathione S-transferases (GST’s) ([Edwards et al., 2000](#_ENREF_14)), WRKY-type transcription factors and WRKY interacting proteins ([Pandey and Somssich, 2009](#_ENREF_43)), wound induced proteins ([Stanford et al., 1989](#_ENREF_56)), Avr9/Cf-9 elicited proteins ([Durrant et al., 2000](#_ENREF_13)), biotic cell death-associated proteins ([Suh et al., 2003](#_ENREF_57)), late embryogenesis abundant or dehydrin-like proteins ([Rorat, 2006](#_ENREF_50)) (Supplementary Table S8) and novel allergen-like proteins of potato (Supplementary Table S9). The largest group of down regulated transcripts encoded protease inhibitors ([Gyetvai et al., 2012](#_ENREF_21)). Three transcripts were annotated as *R* genes (CV492642, TC224349, TC206646) and two as *MLO1* homologues of the barley powdery mildew resistance gene *Mlo* ([Büschges et al., 1997](#_ENREF_5)) (TC209312, TC210948). They were down regulated after infection except TC206646.

The 91 stress related transcripts differentially expressed between genotype pools (Table 3, Supplementary Tables S10 and S11) included 46 that were also consistently up or down regulated after infection (Supplementary Table S12). Fifty three transcripts (58%) showed the MCR conform expression pattern prior to infection (T0) and fourteen showed it at all three time points. Particularly clear examples for differences between genotypic groups were ‘WRKY transcription factor 6’ (TC200826), ‘basic PR-1 protein’ (TC200987), ‘respiratory burst oxidase homologous protein C’ (TC201306), ‘Avr9/Cf-9 rapidly elicited protein 20’(TC206024 and TC214234), a ‘senescence-associated protein’ (TC206431) and a ‘class I chitinase’ (TC222828). Despite the fact that around 200 tags matched to 90 loci encoding *R* genes or *R* gene-like genes (Supplementary Table S2), there were only two *R* gene-like transcripts that fulfilled the filtering criteria for differential expression between genotype pools (TC224349 matching to several members of a clustered family of NBS-LRR resistance proteins (Supplementary Table S10) and tag 49 in Supplementary Table S11). TC224349 was higher expressed in pool A2 than in both pools A1 and B2, whereas tag 49 showed the inverse expression pattern.

**3.3. Chloroplast processes, photosynthesis, carbon fixation.** The third largest group comprised transcripts encoding proteins located in the chloroplast, mostly functional in photosynthesis (e.g. chlorophyll a/b binding proteins, plastocyanin, components of photosystem I and II; oxygen-evolving enhancer protein, ferredoxin), carbon fixation (e.g. ribulose bisphosphate carboxylase (RuBisCo) small chain, RuBisCo activase, carbonic anhydrase) and chloroplastic protein synthesis (50S and 30S ribosomal proteins). Eighty nine percent of these transcripts were down regulated after infection with *P. infestans* (Table 3, Supplementary Tables S8 andS9). This reaction has been observed in diverse plant host-pathogen interactions and is therefore a universal response to biotic stress ([Bilgin et al., 2010](#_ENREF_3)). Notable exceptions were transcripts for beta-carbonic anhydrase (TC214909) and enzymes of the shikimate pathway (TC196130, TC210331, TC211153, see also below). Interestingly, 60 and 63 of 107 transcripts differentially expressed between genotype pools were expressed at higher level in pool A2 compared to both pools A1 and B2 before infection (T0) and two days after infection (T2), respectively. This indicated that A2 plants had a higher steady state level of the majority of transcripts for chloroplast proteins prior to infection and a slower rate of chloroplast decay after infection, consistent with the reduced disease progression in A2 plants. Typical examples for this expression pattern are Oxygen-evolving enhancer protein 1 (TC196189), photosystem I-H protein (TC207691), 50S ribosomal protein L11 (TC202666) and a leucine zipper protein with a putative function in chlorophyll biosynthesis (TC219291) ([Liu et al., 2004](#_ENREF_34))(Supplementary Tables S10 and S11).

**3.4. Membrane and transport proteins.** This rather heterogeneous group included, among others, transcripts related to vesicle-mediated transport ([Thordal-Christensen, 2009](#_ENREF_61)), water and ion channels ([Maurel and Chrispeels, 2001](#_ENREF_38);[Ward et al., 2009](#_ENREF_69)), ABC and major facilitator superfamily transporters ([Pao et al., 1998](#_ENREF_44);[Kang et al., 2011](#_ENREF_27)). The majority of the differential transcripts in this group were up regulated upon infection (Table 3, Supplementary Tables S8, S9). Eight transcripts showed the MCR conform expression pattern at all time points, two of those, annotated as ‘clathrin assembly protein AP17-like protein’([Roca et al., 1998](#_ENREF_48)), in opposite direction (AM907699, TC204068, Supplementary Table S10). Interestingly, both transcripts mapped to the same locus in the potato genome (PGSC0003DMG400009862 on chromosome IV, Figure 5), indicating that the two transcripts corresponded to alleles, one of which was expressed at higher level in pools A1 and B2 and the other in pool A2.

**3.5. Transcription.** This group consisted mainly of putative transcription factors and few nuclear components of RNA synthesis and processing such as RNA polymerase subunits and high-mobility group (HMG) proteins ([Grasser, 1995](#_ENREF_19)). Transcription factors are essential components of defense signaling pathways. Most transcription factors belonged to the Zinc-finger family ([Takatsuji, 1998](#_ENREF_59)) including WRKY transcription factors (see above), followed by the AP2-EREBP family ([Dietz et al., 2010](#_ENREF_10)) including ethylene response factors, the MYB family ([Dubos et al., 2010](#_ENREF_12)) and the Aux/IAA family ([Dargeviciute et al., 1998](#_ENREF_9)). Most transcription factors were up regulated upon infection (Supplementary Tables S8, S9). Promising candidates with an MCR conform expression pattern at all time points are ‘RNA Binding Protein 47’(TC210610) possibly functional in RNA splicing ([Lorković et al., 2000](#_ENREF_35)), a ‘MADS transcription factor’ (TC209814) and ‘AP2/ERF-domain protein’ (TC194984) (Supplementary Table S10).

**3.6. Protein biosynthesis.** This group included mainly ribosomal proteins, translation initiation and elongation factors, and some nuclear factors. ‘Protein biosynthesis’ transcripts in the chloroplast were down regulated upon infection (see above), whereas endoplasmatic protein biosynthesis (e.g. 60S and 40S ribosomal proteins) was up regulated (Supplementary Table S8, S9). Elongation factor 1-alpha, which is frequently used for normalization of transcript levels in qRT-PCR ([Czechowski et al., 2005](#_ENREF_8);[Exposito-Rodriguez et al., 2008](#_ENREF_16)) was also up regulated upon infection (TC209200, TC215306). This transcript should therefore be avoided as reference in comparisons of transcript levels in host-pathogen interactions. Good examples for transcripts showing the MCR conform expression pattern were annotated as ‘60S ribosomal protein L38’ (CK854004), ‘ribosomal protein L3-like’ (TC196036), ‘alpha chain of nascent polypeptide associated complex’ (TC198532, TC216002) and ‘60S ribosomal protein L6’(TC198860) (Supplementary Table S10, S11).

**3.7. Protein degradation**: Transcripts for proteases ([van der Hoorn, 2008](#_ENREF_63)) and components of the ubiquitin-26S proteasome system ([Vierstra, 2009](#_ENREF_65);[Santner and Estelle, 2010](#_ENREF_51)) were grouped under ‘protein degradation’. Three quarters of ‘protein degradation’ transcripts were up regulated upon infection (Table 3, Supplementary Table S8, S9). Clear examples for the MCR conform expression pattern were a subtilisin-like protease (TC207534), wound-inducible carboxypeptidase (TC215914), a polyubiquitin (TC199656), an ubiquitin-conjugating enzyme E2 (TC205158) and an ubiquitin carrier protein (TC218197) (Supplementary Table S10).

**3.8. Protein conformation, chaperone***.* This category included peptidyl-prolyl cis-trans isomerases (PPI’s)([Shaw, 2007](#_ENREF_54)), heat shock proteins ([Wang et al., 2004](#_ENREF_68)) and dehydrins ([Hanin et al., 2011](#_ENREF_22)), which were mostly up regulated in response to infection (Table 3, Supplementary Table S8, S9). The MCR conform expression pattern at all time points was observed for the heat shock proteins HSP70 (TC195822), which was up regulated faster in pool A2 than in both pools A1 and B2, and a DnaJ-like protein isoform (also known as HSP40)(TC204355), which was down regulated and present at lower level in pool A2 compared to A1 and B2 (Supplementary Table S10, S11).

**3.9. Carbohydrate metabolism***.* Sugars play significant roles in host- pathogen interactions as sources of carbon and energy and/or as signaling molecules. They also interact with hormones ([Bolouri Moghaddam and Van den Ende, 2012](#_ENREF_4)). Transcripts for starch biosynthetic enzymes (e. g. glucose-1-phosphate adenylyltransferase, starch synthases), for fructose-bisphosphate aldolase and fructose-1,6-bisphosphatase functional in the Calvin cycle and in gluconeogenesis were mostly down regulated. Transcripts for sugar metabolizing enzymes (e. g. beta-fructofuranosidase/invertase, fructokinase, sucrose synthase, trehalose 6-phosphate synthase, UDP-Glc-4-epimerase), sugar transporters, glycosyl transferases and transcripts for enzymes of the pentose phosphate pathway ([Kruger and von Schaewen, 2003](#_ENREF_31)) were up regulated (Supplementary Table S8). The plastidic pentose phosphate pathway provides precursors for the biosynthesis of aromatic amino acids (shikimic acid pathway) and the subsequent phenylpropanoid pathway. Eight transcripts showed differential expression between genotype pools, one of which was a hexose transporter (TC204383) showing the MCR conform expression pattern at all time points (Supplementary Table S10).

**3.10. Secondary metabolism***.* Plants synthesize a vast array of antimicrobial compounds ([Dixon, 2001](#_ENREF_11)). Several of the relevant pathways were represented by differential transcripts. The group comprised transcripts for enzymes of the mevalonate pathway (mostly up regulated) and the subsequent biosynthesis of steroids, carotenoids and terpenoids ([Lichtenthaler et al., 1997](#_ENREF_33)), of the phenylpropanoid pathway and lignin, alkaloids, flavonoids and anthocyanins derived from it ([Vogt, 2010](#_ENREF_66)), and for 2-oxoglutarate-dependent dioxygenase (down regulated) involved in glucosinolate biosynthesis([Sønderby et al., 2010](#_ENREF_55)). Interesting examples for the MCR conform expression pattern were a Caffeoyl-CoA O-methyltransferase (TC223431) that was absent in the A2 pool but present at low level in pools A1 and B2 before and one day after infection, and a putative (S)-N-methylcoclaurine 3'-hydroxylase (TC215758)([Pauli and Kutchan, 1998](#_ENREF_45)) expressed at lower level in the A2 pool than in pools A1 and B2 (Supplementary Tables S8, S9, S10, S11).

**3.11. Cell wall***.* Differential transcripts in this group were annotated as structural cell wall proteins and cell wall biosynthetic or degrading enzymes ([Cassab, 1998](#_ENREF_6);[Keegstra, 2010](#_ENREF_28)), including one of the most frequent transcripts for a glycine-rich cell wall protein ([Gyetvai et al., 2012](#_ENREF_21)). The majority of these transcripts were down regulated, for example most proline-rich and arabinogalactan proteins ([Ellis et al., 2010](#_ENREF_15)), cell wall degrading xyloglucan endotransglucosylase-hydrolases (XTH) ([Miedes and Lorences, 2007](#_ENREF_39)) and cellulose synthases ([Richmond and Somerville, 2000](#_ENREF_47)), while most extensins ([Lamport et al., 2011](#_ENREF_32)) were up regulated (Supplementary Tables S8, S9). The MCR conform expression pattern at all time points showed an extensin (TC222646) and a pectin methyl esterase (TC226210), which was absent in pool A2 but present in both pools B2 and A1 (Supplementary Table S10).

**3.12. Development, regulation, signaling.** Transcripts with the most clear functional annotation in this group were coding for protein kinases ([Romeis, 2001](#_ENREF_49)) including a SNF1-related kinase (TC208530) and protein phosphatase 2C (PP2C, TC199745, TC219360, Supplementary Table S8, S9). SNF1 and PP2C have been established as components of early abscisic acid (ABA) signaling ([Hubbard et al., 2010](#_ENREF_24)). Interestingly, the transcript for SNF1-related kinase was consistently expressed at lower level in pool A2 than in both pools A1 and B2 (Supplementary Table S10 ).

**3.13. Lipid metabolism.** Lipids and lipid signaling are important components in host- pathogen interactions ([Shah, 2005](#_ENREF_53);[Christensen and Kolomiets, 2011](#_ENREF_7)). Upon infection differentially expressed were, for example, transcripts for lipoxygenases (LOX) and dioxygenases (DOX) (both up regulated), several desaturases, lipases and lipid transfer proteins ([García-Olmedo et al., 1995](#_ENREF_18))(up or down regulated). One LOX transcript (TC204989) was expressed at higher level at T0 in pool A2 and was then strongly and transiently induced at T1, whereas induction was delayed in pools A1 and B2. A transcript for ‘biotin carboxylase carrier protein’ (TC196756) ([Nikolau et al., 2003](#_ENREF_41)) was expressed at higher level in pool A2 than in pools A1 and B2 at all time points (Supplementary Table S10).

**3.14. Central metabolism.** This group comprised predominantly up regulated transcripts for proteins functional in the mitochondrial respiration chain (e. g. subunits of NADH-ubiquinone oxidoreductase, ATP synthase, cytochrome c oxidase), in the tricarboxylic acid (TCA) and glyoxylate cycle (e. g. isocitrate dehydrogenase [NADP^+^]), and transcripts for proteins functional in glycolysis or Calvin cycle (e. g. Glyceraldehyde-3-phosphate dehydrogenase), which were regulated in both directions, up and down. Isocitrate dehydrogenase [NADP^+^] (TC206316, Supplementary Table S10), which appears to be induced by leaf senescence ([Gut and Matile, 1988](#_ENREF_20)), was more strongly up regulated in pools A1 and B2 compared with A2, consistent with slower senescence of the plants in pool A2 caused by *P. infestans* infection. A ‘glyceraldehyde 3-phosphate dehydrogenase-like protein’ (TC223368, Supplementary Table S10) and ‘ubiquinol-cytochrome c reductase complex 6.7 kDa protein’ (tag 156 in Supplementary Table S11) ([Jänsch et al., 1995](#_ENREF_26)) also showed the MCR conform expression pattern at all time points.

**3.15. Hormone metabolism and signaling.** Plant hormones play a key role in defense signaling. Transcripts functional in ethylene signaling ([Wang et al., 2002](#_ENREF_67)) were the most frequent in this group, followed by components of auxin ([Fu and Wang, 2011](#_ENREF_17)), jasmonate ([Kombrink, 2012](#_ENREF_29)), giberellic acid ([Schwechheimer and Willige, 2009](#_ENREF_52)), salicylic acid ([An and Mou, 2011](#_ENREF_2)), cytokinin ([Hwang et al., 2012](#_ENREF_25)) and abscisic acid ([Ton et al., 2009](#_ENREF_62)) signaling pathways. With one exception (EIN3-binding F-box protein 1, TC217823) transcripts functional in ethylene signaling were up regulated, whereas the two transcripts linked to the salicylic acid pathway (TC218774, TC222167) were down regulated (Supplementary Table S8). In particular, components of jasmonate signaling were jasmonate ZIM-domain proteins (JAZ) 1 (TC205215, TC208021) and 3 (TC204966), prosystemin (TC208562)([Sun et al., 2011](#_ENREF_58)) and fatty acid hydroperoxide lyase (TC202396)([Noordermeer et al., 2001](#_ENREF_42)). Interestingly, the two transcripts for JAZ 1 were allelic, but responded to infection in opposite direction. TC205215 was down, whereas TC208021 was up regulated. Prosystemin was up regulated at T1, but less so in pool A2 than in pools A1 and B2 (Supplementary Table S8). JAZ 1 and JAZ 3 showed the MCR conform expression pattern in uninfected plants, with higher transcript levels in pool A2 compared to both pools A1 and B2 (Figure 6D and Supplementary Table S10). Fatty acid hydroperoxide lyase was expressed at very low level in pools A1 and B2 but was absent in pool A2 (Supplementary Table S10). The most remarkable transcripts showing the MCR conform expression pattern at all infection time points were an AP2/ERF-domain containing putative transcription factor (TC194984), an ethylene receptor homolog (tag 157 in Supplementary Table S11), both expressed at lower level in the A2 pool compared to pools A1 and B2, and ‘salicylic acid-binding protein 2’ (TC222167), which showed the opposite expression pattern (Supplementary Table S10).

**3.16. Amino acid metabolism***.* In this category were, among others, transcripts for enzymes of the shikimate pathway and aromatic amino acid biosynthesis (DN590166, TC195056, TC195989, TC196130, TC210331, TC211153, TC220850 in Supplementary Table S8, tag 25 in Supplementary Table S9), which provide the precursors for auxin and salicylic acid, for cell wall components, alkaloids and many other secondary metabolites ([Maeda and Dudareva, 2012](#_ENREF_37)). With one exception (TC195056) these transcripts were strongly up regulated, despite the fact that the shikimate pathway is located in the chloroplast. None of them differed consistently between genotype pools. The MCR conform expression pattern was observed at all time points for a putative aspartate aminotransferase (TC198566), asparagine synthetase (TC212265) and acetolactate synthase (TC206045) (Supplementary Table S10).

**3.17. Other***.* The transcripts in this large and interesting group were predominantly up regulated after infection (Table 3). Most conspicuous were transcripts annotated as cytochrome P450 proteins having oxidative functions in multiple pathways ([Werck-Reichhart and Feyereisen, 2000](#_ENREF_70)), other oxidoreductases, proteins involved in chromatin remodeling as part of stress responses ([Luo et al., 2012](#_ENREF_36)) such as histones and methyltransferases (e. g. DR034792, TC215220), components of the cytoskeleton such as actin, tubulin and myosin-related proteins ([Kost and Chua, 2002](#_ENREF_30);[Takemoto and Hardham, 2004](#_ENREF_60)), several calmodulins ([Reddy et al., 2011](#_ENREF_46)), metal ion and nucleic acid binding proteins and allergen-like proteins, which may be novel PR proteins ([Hoffmann-Sommergruber, 2000](#_ENREF_23)). Examples for transcripts with consistently higher expression levels in pool A2 than in both pools A1 and B2 were ‘ATP-dependent DNA helicase RecG’ (DN589479), ‘globulin’ (TC207606) and ‘small nuclear ribonucleoprotein-like protein’ (TC208186). The inverse pattern, lower expression in pool A2 than in pools A1 and B2, were most clearly shown by ‘squalene monooxygenase’(AM906901), ‘major latex’ protein (TC210217) ([Nessler, 1988](#_ENREF_40)), a calmodulin (tag 2 in Supplementary Table S10) and an ‘ATP binding protein’ (tag 60 in Supplementary Table S10)(Supplementary Table S9).

**References**

Almagro, L., Gómez Ros, L.V., Belchi-Navarro, S., Bru, R., Ros Barceló, A., and Pedreño, M.A. (2009). Class III peroxidases in plant defence reactions. *Journal of Experimental Botany* 60**,** 377-390.

An, C., and Mou, Z. (2011). Salicylic acid and its function in plant Immunity. *Journal of Integrative Plant Biology* 53**,** 412-428.

Bilgin, D.D., Zavala, J.A., Zhu, J.I.N., Clough, S.J., Ort, D.R., and Delucia, E.H. (2010). Biotic stress globally downregulates photosynthesis genes. *Plant, Cell & Environment* 33**,** 1597-1613.

Bolouri Moghaddam, M.R., and Van Den Ende, W. (2012). Sugars and plant innate immunity. *Journal of Experimental Botany*.

Büschges, R., Hollricher, K., Panstruga, R., Simons, G., Wolter, M., Frijters, A., Van Daelen, R., Van Der Lee, T., Diergaarde, P., Groenendijk, J., Töpsch, S., Vos, P., Salamini, F., and Schulze-Lefert, P. (1997). The barley *Mlo* gene: A novel control element of plant pathogen resistance. *Cell* 88**,** 695-705.

Cassab, G.I. (1998). Plant cell wall proteins. *Annu. Rev. Plant Physiol. Plant Mol. Biol.* 49**,** 281-309.

Christensen, S.A., and Kolomiets, M.V. (2011). The lipid language of plant–fungal interactions. *Fungal Genetics and Biology* 48**,** 4-14.

Czechowski, T., Stitt, M., Altmann, T., Udvardi, M.K., and Scheible, W.-R. (2005). Genome-wide identification and testing of superior reference genes for transcript normalization in Arabidopsis. *Plant Physiol* 139**,** 5-17.

Dargeviciute, A., Roux, C., Decreux, A., Sitbon, F., and Perrot-Rechenmann, C. (1998). Molecular cloning and expression of the early auxin-responsive Aux/IAA gene family in *Nicotiana tabacum*. *Plant and Cell Physiology* 39**,** 993-1002.

Dietz, K.-J., Vogel, M., and Viehhauser, A. (2010). AP2/EREBP transcription factors are part of gene regulatory networks and integrate metabolic, hormonal and environmental signals in stress acclimation and retrograde signalling. *Protoplasma* 245**,** 3-14.

Dixon, R.A. (2001). Natural products and plant disease resistance. *Nature* 411**,** 843-847.

Dubos, C., Stracke, R., Grotewold, E., Weisshaar, B., Martin, C., and Lepiniec, L. (2010). MYB transcription factors in *Arabidopsis*. *Trends in Plant Science* 15**,** 573-581.

Durrant, W.E., Rowland, O., Piedras, P., Hammond-Kosack, K.E., and Jones, J.D.G. (2000). cDNA-AFLP reveals a striking overlap in race-specific resistance and wound response gene expression profiles. *The Plant Cell Online* 12**,** 963-977.

Edwards, R., Dixon, D.P., and Walbot, V. (2000). Plant glutathione S-transferases: enzymes with multiple functions in sickness and in health. *Trends in Plant Science* 5**,** 193-198.

Ellis, M., Egelund, J., Schultz, C.J., and Bacic, A. (2010). Arabinogalactan-proteins: Key regulators at the cell surface? *Plant Physiology* 153**,** 403-419.

Exposito-Rodriguez, M., Borges, A., Borges-Perez, A., and Perez, J. (2008). Selection of internal control genes for quantitative real-time RT-PCR studies during tomato development process. *BMC Plant Biology* 8**,** 131.

Fu, J., and Wang, S. (2011). Insights into auxin signaling in plant–pathogen interactions. *Frontiers in Plant Science* 2.

García-Olmedo, F., Molina, A., Segura, A., and Moreno, M. (1995). The defensive role of nonspecific lipid-transfer proteins in plants. *Trends in Microbiology* 3**,** 72-74.

Grasser, K.D. (1995). Plant chromosomal high mobility group (HMG) proteins. *The Plant Journal* 7**,** 185-192.

Gut, H., and Matile, P. (1988). Apparent induction of key enzymes of the glyoxylic acid cycle in senescent barley leaves. *Planta* 176**,** 548-550.

Gyetvai, G., Sønderkær, M., Göbel, U., Basekow, R., Ballvora, A., Imhoff, M., Kersten, B., Nielsen, K.-L., and Gebhardt, C. (2012). The transcriptome of compatible and incompatible interactions of potato (*Solanum tuberosum*) with *Phytophthora infestans* revealed by DeepSAGE analysis. *PLoS ONE* 7**,** e31526.

Hanin, M., Brini, F., Ebel, C., Toda, Y., Takeda, S., and Masmoudi, K. (2011). Plant dehydrins and stress tolerance: versatile proteins for complex mechanisms. *Plant Signaling & Behavior* 6**,** 1503-1509.

Hoffmann-Sommergruber, K. (2000). Plant allergens and pathogenesis-related proteins. *International Archives of Allergy and Immunology* 122**,** 155-166.

Hubbard, K.E., Nishimura, N., Hitomi, K., Getzoff, E.D., and Schroeder, J.I. (2010). Early abscisic acid signal transduction mechanisms: newly discovered components and newly emerging questions. *Genes & Development* 24**,** 1695-1708.

Hwang, I., Sheen, J., and Müller, B. (2012). Cytokinin signaling networks. *Annual Review of Plant Biology* 63**,** 353-380.

Jänsch, L., Kruft, V., Schmitz, U.K., and Braun, H.-P. (1995). Cytochrome c reductase from potato does not comprise three core proteins but contains an additional low-molecular-mass subunit. *European Journal of Biochemistry* 228**,** 878-885.

Kang, J., Park, J., Choi, H., Burla, B., Kretzschmar, T., Lee, Y., and Martinoia, E. (2011). Plant ABC Transporters. *The Arabidopsis Book***,** e0153.

Keegstra, K. (2010). Plant Cell Walls. *Plant Physiology* 154**,** 483-486.

Kombrink, E. (2012). Chemical and genetic exploration of jasmonate biosynthesis and signaling paths. *Planta* 236**,** 1351-1366.

Kost, B., and Chua, N.-H. (2002). The plant cytoskeleton: Vacuoles and cell walls make the difference. *Cell* 108**,** 9-12.

Kruger, N.J., and Von Schaewen, A. (2003). The oxidative pentose phosphate pathway: structure and organisation. *Current Opinion in Plant Biology* 6**,** 236-246.

Lamport, D.T.A., Kieliszewski, M.J., Chen, Y., and Cannon, M.C. (2011). Role of the extensin superfamily in primary cell wall architecture. *Plant Physiology* 156**,** 11-19.

Lichtenthaler, H.K., Rohmer, M., and Schwender, J. (1997). Two independent biochemical pathways for isopentenyl diphosphate and isoprenoid biosynthesis in higher plants. *Physiologia Plantarum* 101**,** 643-652.

Liu, N., Yang, Y.-T., Liu, H.-H., Yang, G.-D., Zhang, N.-H., and Zheng, C.C. (2004). NTZIP antisense plants show reduced chlorophyll levels. *Plant Physiology and Biochemistry* 42**,** 321-327.

Lorković, Z.L., Wieczorek Kirk, D.A., Klahre, U., Hemmings-Mieszczak, M., and Filipowicz, W. (2000). RBP45 and RBP47, two oligouridylate-specific hnRNP-like proteins interacting with poly(A)+ RNA in nuclei of plant cells. *RNA* 6**,** 1610-1624.

Luo, M., Liu, X., Singh, P., Cui, Y., Zimmerli, L., and Wu, K. (2012). Chromatin modifications and remodeling in plant abiotic stress responses. *Biochimica et Biophysica Acta (BBA) - Gene Regulatory Mechanisms* 1819**,** 129-136.

Maeda, H., and Dudareva, N. (2012). The shikimate pathway and aromatic amino acid biosynthesis in plants. *Annual Review of Plant Biology* 63**,** 73-105.

Maurel, C., and Chrispeels, M.J. (2001). Aquaporins. A molecular entry into plant water relations. *Plant Physiology* 125**,** 135-138.

Miedes, E., and Lorences, E.P. (2007). The implication of xyloglucan endotransglucosylase/hydrolase (XTHs) in tomato fruit infection by *Penicillium expansum* Link. A. *Journal of Agricultural and Food Chemistry* 55**,** 9021-9026.

Nessler, C.L. (1988). Comparative analysis of the major latex proteins of opium poppy. *Journal of Plant Physiology* 132**,** 588-592.

Nikolau, B.J., Ohlrogge, J.B., and Wurtele, E.S. (2003). Plant biotin-containing carboxylases. *Archives of Biochemistry and Biophysics* 414**,** 211-222.

Noordermeer, M.A., Veldink, G.A., and Vliegenthart, J.F.G. (2001). Fatty Acid Hydroperoxide Lyase: A Plant Cytochrome P450 Enzyme Involved in Wound Healing and Pest Resistance. *ChemBioChem* 2**,** 494-504.

Pandey, S.P., and Somssich, I.E. (2009). The role of WRKY transcription factors in plant immunity. *Plant Physiology* 150**,** 1648-1655.

Pao, S.S., Paulsen, I.T., and Saier, M.H.J. (1998). Major facilitator superfamily. *Microbiology and Molecular Biology Reviews* 62**,** 1-34.

Pauli, H.H., and Kutchan, T.M. (1998). Molecular cloning and functional heterologous expression of two alleles encoding (S)-N-methylcoclaurine 3′-hydroxylase (CYP80B1), a new methyl jasmonate-inducible cytochrome P-450-dependent mono-oxygenase of benzylisoquinoline alkaloid biosynthesis. *The Plant Journal* 13**,** 793-801.

Reddy, A.S.N., Ali, G.S., Celesnik, H., and Day, I.S. (2011). Coping with stresses: Roles of Calcium- and Calcium/calmodulin-regulated gene expression. *The Plant Cell Online* 23**,** 2010-2032.

Richmond, T.A., and Somerville, C.R. (2000). The cellulose synthase superfamily. *Plant Physiology* 124**,** 495-498.

Roca, R., Stiefel, V., and Puigdomènech, P. (1998). Characterization of the sequence coding for the clathrin coat assembly protein AP17 (σ2) associated with the plasma membrane from Zea mays and constitutive expression of its gene. *Gene* 208**,** 67-72.

Romeis, T. (2001). Protein kinases in the plant defence response. *Current Opinion in Plant Biology* 4**,** 407-414.

Rorat, T. (2006). Plant dehydrins — Tissue location, structure and function. *Cellular & Molecular Biology Letters* 11**,** 536-556.

Santner, A., and Estelle, M. (2010). The ubiquitin-proteasome system regulates plant hormone signaling. *The Plant Journal* 61**,** 1029-1040.

Schwechheimer, C., and Willige, B.C. (2009). Shedding light on gibberellic acid signalling. *Current Opinion in Plant Biology* 12**,** 57-62.

Shah, J. (2005). Lipids, lipases, and lipid-modifying enzymes in plant disease resistance. *Annual Review of Phytopathology* 43**,** 229-260.

Shaw, P.E. (2007). Peptidyl-prolyl cis/trans isomerases and transcription: is there a twist in the tail? *EMBO Rep* 8**,** 40-45.

Sønderby, I.E., Geu-Flores, F., and Halkier, B.A. (2010). Biosynthesis of glucosinolates – gene discovery and beyond. *Trends in Plant Science* 15**,** 283-290.

Stanford, A., Bevan, M., and Northcote, D. (1989). Differential expression within a family of novel wound-induced genes in potato. *Mol Gen Genet* 215**,** 200-2008.

Suh, M.C., Oh, S.-K., Kim, Y.-C., Pai, H.-S., and Choi, D. (2003). Expression of a novel tobacco gene, NgCDM1, is preferentially associated with pathogen-induced cell death. *Physiological and Molecular Plant Pathology* 62**,** 227-235.

Sun, J.-Q., Jiang, H.-L., and Li, C.-Y. (2011). Systemin/Jasmonate-Mediated Systemic Defense Signaling in Tomato. *Molecular Plant* 4**,** 607-615.

Takatsuji, H. (1998). Zinc-finger transcription factors in plants. *Cell. Mol. Life Sci.* 54**,** 582-596.

Takemoto, D., and Hardham, A.R. (2004). The cytoskeleton as a regulator and target of biotic interactions in plants. *Plant Physiology* 136**,** 3864-3876.

Thordal-Christensen, H. (2009). "Vesicle trafficking in plant pathogen defence," in *Signaling in Plants, Signaling and Communication in Plants,* eds. F. Baluska & S. Mancusco. (Berlin Heidelberg: Springer), 287-301.

Ton, J., Flors, V., and Mauch-Mani, B. (2009). The multifaceted role of ABA in disease resistance. *Trends in Plant Science* 14**,** 310-317.

Van Der Hoorn, R.a.L. (2008). Plant proteases: From phenotypes to molecular mechanisms. *Annual Review of Plant Biology* 59**,** 191-223.

Van Loon, L.C., Rep, M., and Pieterse, C.M.J. (2006). Significance of inducible defense-related proteins in infected plants. *Annual Review of Phytopathology* 44**,** 135-162.

Vierstra, R.D. (2009). The ubiquitin-26S proteasome system at the nexus of plant biology. *Nat Rev Mol Cell Biol* 10**,** 385-397.

Vogt, T. (2010). Phenylpropanoid biosynthesis. *Molecular Plant* 3**,** 2-20.

Wang, K.L.-C., Li, H., and Ecker, J.R. (2002). Ethylene Biosynthesis and Signaling Networks. *The Plant Cell Online* 14**,** S131-S151.

Wang, W., Vinocur, B., Shoseyov, O., and Altman, A. (2004). Role of plant heat-shock proteins and molecular chaperones in the abiotic stress response. *Trends in Plant Science* 9**,** 244-252.

Ward, J.M., Mäser, P., and Schroeder, J.I. (2009). Plant ion channels:Gene families, physiology, and functional genomics analyses. *Annu Rev Physiol* 71**,** 59-82.

Werck-Reichhart, D., and Feyereisen, R. (2000). Cytochromes P450: a success story. *Genome Biology* 1**,** reviews3003.3001 - reviews3003.3009.
